# Copy number variation is highly correlated with differential gene expression: a pan-cancer study

**DOI:** 10.1186/s12881-019-0909-5

**Published:** 2019-11-09

**Authors:** Xin Shao, Ning Lv, Jie Liao, Jinbo Long, Rui Xue, Ni Ai, Donghang Xu, Xiaohui Fan

**Affiliations:** 1 College of Pharmaceutical Sciences, Zhejiang University, Hangzhou, 310058 China; 2 Department of Pharmacy, The 2nd Affiliated Hospital, School of Medicine, Zhejiang University, Hangzhou, 310009 China

**Keywords:** Copy number variation, Differential gene expression, Concordance, Pan-cancer

## Abstract

**Background:**

Cancer is a heterogeneous disease with many genetic variations. Lines of evidence have shown copy number variations (CNVs) of certain genes are involved in development and progression of many cancers through the alterations of their gene expression levels on individual or several cancer types. However, it is not quite clear whether the correlation will be a general phenomenon across multiple cancer types.

**Methods:**

In this study we applied a bioinformatics approach integrating CNV and differential gene expression mathematically across 1025 cell lines and 9159 patient samples to detect their potential relationship.

**Results:**

Our results showed there is a close correlation between CNV and differential gene expression and the copy number displayed a positive linear influence on gene expression for the majority of genes, indicating that genetic variation generated a direct effect on gene transcriptional level. Another independent dataset is utilized to revalidate the relationship between copy number and expression level. Further analysis show genes with general positive linear influence on gene expression are clustered in certain disease-related pathways, which suggests the involvement of CNV in pathophysiology of diseases.

**Conclusions:**

This study shows the close correlation between CNV and differential gene expression revealing the qualitative relationship between genetic variation and its downstream effect, especially for oncogenes and tumor suppressor genes. It is of a critical importance to elucidate the relationship between copy number variation and gene expression for prevention, diagnosis and treatment of cancer.

## Background

Genetic structural variation in the human genome can be present in many forms, ranging from single nucleotide polymorphisms (SNPs) to large chromosome aberrance [1]. In the past, SNPs are regarded as the predominant form of structural variation and account for much phenotypic variation [2, 3]. However, recent studies show the widespread existence of copy number variation (CNV) in individuals, and since that these observations have been extremely appreciated and expanded [4–6]. In general, CNV is defined as an amplifying or decreasing number of DNA segments that is 1 kb or larger in the human genome [1, 4, 5], which accounts for an important part of genetic structural variation. Currently great efforts in science community have been directed to catalog and characterize somatic CNV in a comprehensive manner [7, 8], which provides key knowledge on how they impact biological function, evolution and human diseases on genomic level.

It is generally accepted that somatic CNV is highly associated with the development and progression of numerous cancers by impacting gene expression level [9–19]. Samulin Erdem et al. [13] found that Neurofascin (NFASC) gene is significantly amplified and overexpressed in non-small cell lung cancer (NSCLC) patients and the novel role of NFASC is identified in the regulation of cell motility and NSCLC migration. Dong et al. [16] analyzed the copy number alterations and differentially transcribed genes in esophageal cancer and observed a noteworthy association between CNV and differential gene expression for FAM60A, TFDP1, CDC25B and MCM2. Subsequently, FAM60A was identified as a potential prognostic factor with a striking correlation to overall survival and clinical-pathological parameters. Lines of evidence support differential gene expression might be a vital intermediate mechanism for CNV to exert effect on the downstream phenotype.

Despite a number of studies have explored CNV and differential gene expression of several classical oncogenes or tumor suppressor genes in different cancers [12, 14, 17, 18, 20], there has been no systematic study about the relationship between CNV and differential gene expression across a broader spectrum of cancer types and cell lines. It is unclear to what extent the expression level is affected by CNV for the whole genomics. Previous observations from single gene or single cancer type may not be representative for other genes or other types of cancer. Here we aimed to systematically investigate the specific relationship between somatic CNV and differential gene expression across cell lines and different cancer types for known genes. This study may help us better understand the correlation between CNV and differential gene expression and provide new insights into the mechanism of development and progression of cancer.

## Methods

### Data sources

The copy number and mRNA expression data of Broad-Novartis Cancer Cell Line Encyclopedia (CCLE), NCI-60 and the Cancer Genome Atlas (TCGA) were collected from the cBioportal for Cancer Genomics (http://www.cbioportal.org/). The cell lines datasets of CCLE and NCI-60 contained 966 and 59 cell lines respectively and the TCGA datasets contained 31 types of cancer of 9159 samples (see Additional file 2: Table S1). Putative copy number calls were determined by using GISTIC 2.0 [21], while expression levels were quantified by RSEM [22] from RNA-Seq data for TCGA. Another independent dataset were curated from COSMIC Cell Lines Project (CCLP, v81, http://cancer.sanger.ac.uk/cell_lines) containing 1020 cell lines. The copy number was obtained from Affymetrix SNP6.0 array data with PICNIC [23].

### Definition of four variation tendencies

For CCLE, NCI60 and TCGA datasets, gene-wise homozygous deletion or high level amplification were regarded as copy number amplified or deleted gene (Copy number values: − 2 = homozygous deletion; − 1 = hemizygous deletion; 0 = neutral / no change; 1 = gain; 2 = high level amplification). For CCLP, copy number amplification was obtained by the following criteria: (the average genome ploidy < =2.7 AND total DNA segment copy number > =5) OR (average genome ploidy > 2.7 AND total DNA segment copy number > =9). While the criteria for copy number deletion was: (the average genome ploidy < =2.7 AND total DNA segment copy number = 0) OR (average genome ploidy > 2.7 AND total DNA segment copy number < (average genome ploidy – 2.7)). Gene expression levels were quantified by RSEM from RNA-Seq data and mRNA Z scores were computed using the tumors samples that are diploid for the corresponding gene. For each gene, Z score = (x-u)/o, where u, o represent the average expression and standard deviation of this gene across samples, respectively; x represents the specific expression of this gene in a specific sample. Differentially expressed genes (DEGs) were further filtered out as Z scores more than 2 (upregulated genes) or less than − 2 (downregulated genes). Thus, the four variation tendencies were defined as follows: amplified AND upregulated; amplified AND downregulated; deleted AND upregulated; deleted AND downregulated.

### Identification of amplified and upregulated genes (AUGs) and deleted and downregulated (DDGs)

copy number amplification and expression level upregulation ratio against copy number deletion and expression level downregulation, copy number deletion and expression level downregulation ratio against copy number amplification and expression level upregulation more than 50 % on each gene across 9159 tumor samples were applied to identify AUGs and DDGs with a higher ρ (> 0.4) and a higher number (> 146.5) of copy number amplification and expression level upregulation than the median level of 30 most popular oncogenes (Additional file 2: Table S4) or a higher ρ (> 0.41) and a higher number (> 18.5) of copy number deletion and expression level downregulation than the median level of 10 tumor suppressor genes (Additional file 2: Table S5). AUGs and DDGs were sorted by the amount of copy number amplification and expression level upregulation, copy number deletion and expression level downregulation respectively and 31 representative AUGs and 29 representative DDGs matched with KEGG genes were identified from top 100 highly concordant genes.

### PPI network construction and functional enrichments

Search Tool for the Retrieval of Interacting Genes (STRING, http://www.string-db.org/) was used to construct the PPI network of FYTTD1. The line thickness indicates the strength of data support from the sources of text mining and experiments with a cutoff value of medium confidence (0.4). Then the functional enrichment results of KEGG pathways and Gene Ontology (GO) biological process were applied to the PPI network with false discovery rate less than 0.05.

### Statistical analyses

R (version 3.4.2) including R packages of *data.table* for data cleaning and management, *survival* for survival analysis, *ggplot2* for data visualization and GraphPad Prism 7.0 were used for the statistical analysis. ρ is equal to the Pearson correlation between the rank values of those two variables to assesses how well the relationship between two variables can be described using a monotonic function and were calculated by the function of cor() in R. Differences between two groups were determined using the Welch’s t-test (significant with *p* < 0.05).

## Results

### Differential gene expression is highly associated with CNV across multiple cancer types and cancer cell lines

**Fig. 1:**
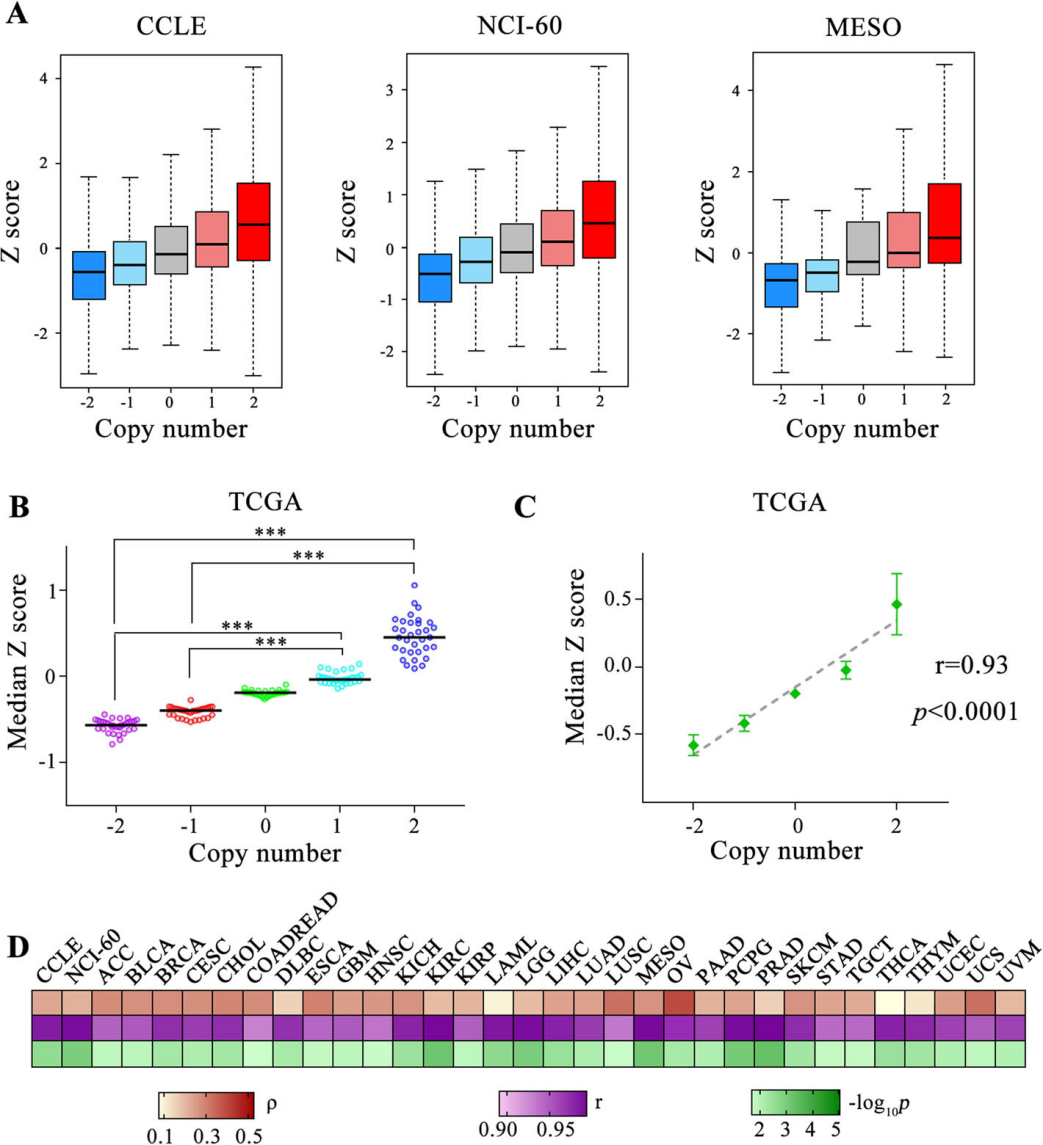
Integrative analysis of the association between CNV and differential gene expression. **a.** Boxplots show the relationship of Z score (y-axis) versus copy number value (x-axis) across 966 cell lines for CCLE, 59 cell lines for NCI-60 and 87 tissue samples for MESO. **b.** Median Z score versus copy number from 31 cancer types was extracted in scatter plot. *P* values were obtained from a Welch’s t-test. *** means *p* < 0.0001.**c.** Linear regression analysis of median Z score versus copy number within 31 tumors. **d**. Heat map shows the ρ between Z scores of gene expression and copy number, Pearson’s r of fitting and *p* value of linear regression analysis on median Z score of gene expression versus copy number among cell lines and tumors. ρ: spearman’s correlation coefficient

It is known that CNV is related to alteration on gene expression. However, the correlations between CNV and gene expression change on a global cellular scale remain to be elucidated. To comprehensively exploit this question, we collected available genomic datasets from the Broad-Novartis Cancer Cell Line Encyclopedia (CCLE), NCI-60, and the Cancer Genome Atlas (TCGA). Integrated analysis of CNV and differential gene expression was performed across 31 cancer types and 2 cancer cell lines resources (Additional file 2: Table S1). Our results showed an apparent effect of copy number on gene expression (Fig. 1). Interestingly, gene-wise copy number amplification appears to harbor a higher median Z score, indicating enhanced gene expression, while copy number deletion usually led to decreased gene expression among 966 cell lines for CCLE datasets (Fig. 1a). Even for NCI-60 project and mesothelioma (MESO) in TCGA, which have smaller sample size, this consistent tendency still is kept (Fig. 1a; results of other 30 cancer types in TCGA found in Additional file 1: Figure S1 and Figure S2). For all cancer types under study, median Z scores of gene expression with copy number amplified were strikingly higher than those with copy number deleted (Fig. 1B). In order to further detect the relation between copy number and transcript level, the linear regression model was taken for each cancer type of TCGA datasets. Fitting results indicated gene expression level was prominently linear-correlated with copy number (*r* = 0.93, *p* < 0.0001; Fig. 1c). In addition, the spearman’s correlation coefficient (ρ) test and linear regression analysis among cell lines and tumors were implemented to uncover the close association between differential gene expression and CNV with ρ ranging from 0.075 to 0.53 (mean *r* = 0.97; Fig. 1d), indicating the positive correlation of differentially transcriptional level and CNV in general.

### Most genes’ expression changes significantly correlated with their CNVs

**Fig. 2:**
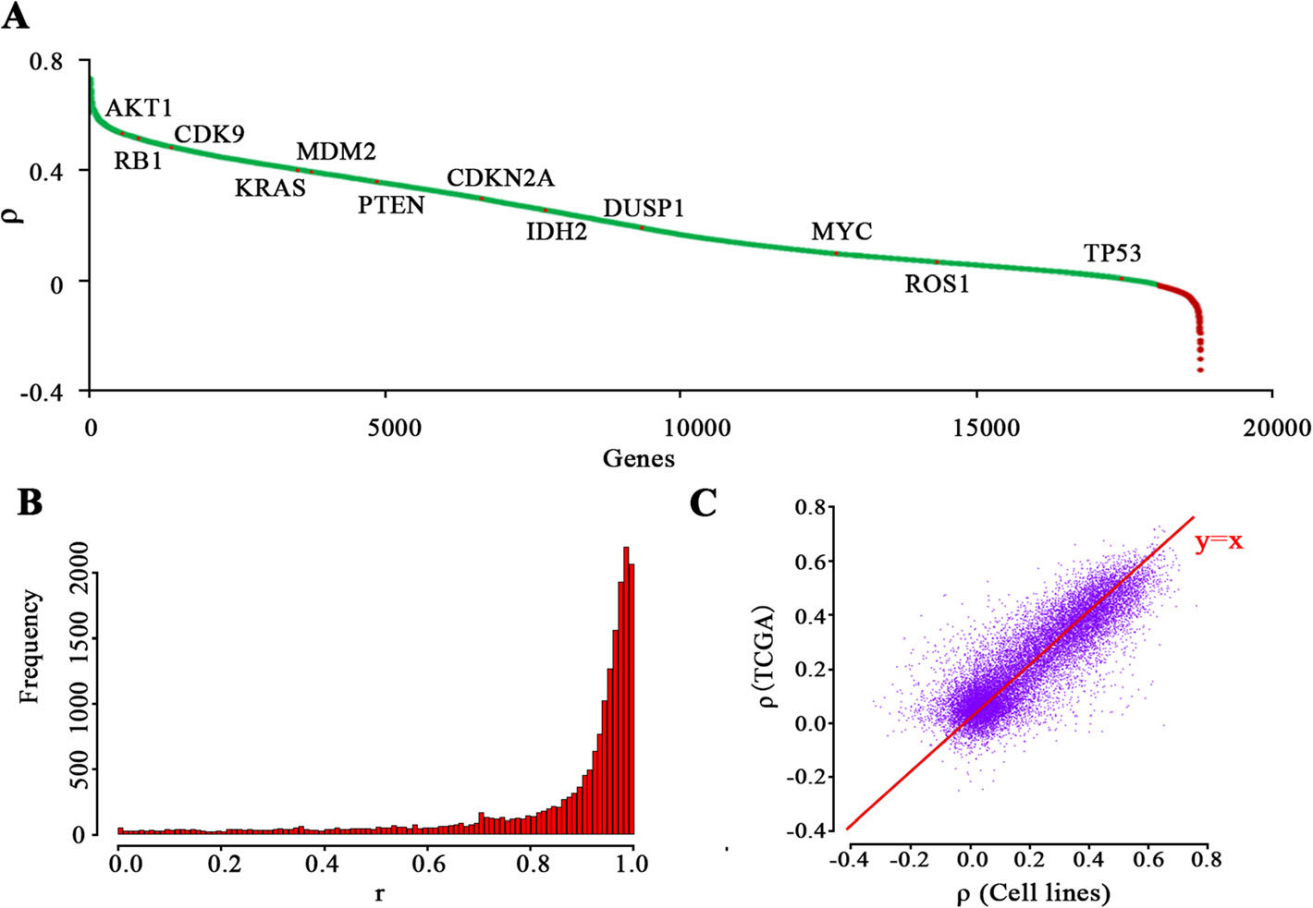
Most genes’ expression changes significantly correlated with their copy number variations. **a.** Genes were sorted according to ρ on Z score versus copy number across 9159 tumor samples from TCGA, green representing a positive ρ and red meaning a negative one, representative oncogenes and tumor suppressor genes next to the corresponding points. **b.** Histograms present the distribution of r for each genes based on the linear fitting results of median Z score with each variable copy number in TCGA datasets, the majority of Pearson’s r of fitting distributed ranging from 0.8 to 1. **c.** A high level of agreement on ρ for 16,639 shared genes between cell lines dataset and TCGA dataset, y-axis for TCGA dataset and x-axis for cell lines dataset. ρ: spearman’s correlation coefficient

We next analyzed the relationship between copy number and expression level at the basis of individual gene. Remarkably, for substantial number of genes, their copy numbers exert a positive correlation with the corresponding gene expression level among 1025 cell lines dataset (88.11%; Additional file 1: Figure S3A; Figure S3B) and 9159 tumor samples (94.15%; Fig. 2a). This trend is particularly obvious for a vast number of oncogenes (e.g. MYC, AKT1, CDK9, KRAS, and MDM2) and tumor suppressor genes (e.g. CDKN2A, RB1, PTEN, and TP53). Linear regression was taken to explore whether copy number had a positive linear influence on gene expression. Plenty of genes presented a high degree of fitting with r intensively ranging from 0.8 to 1 (Fig. 2b; Additional file 1: Figure S3C), genomic structural variation may generate a direct effect on gene transcriptional level for the majority of genes. Concordantly, a recent study by GTEx consortium also associated genetic variants with gene expression levels across 44 human healthy tissues and gene expression levels were found to be affected by local genetic variation for most genes based on eQTL analysis [24]. In addition, we selected 10 genes with a significant correlation between CNV and differential gene expression through TCGA and cell lines datasets and validated this relationship through literature mining. For 9 genes except the weak evidence for NFASC, a strong correlation between CNV and differential gene expression was confirmed combining ρ and Pearson’s r of fitting (Table 1), in accordance with previous experimental findings.

**Table 1:** Correlation validation of genes with a significant correlation between CNV and differential gene expression in literature

Official symbol	ρ Cell lines	r Cell lines	ρ TCGA	r TCGA	Reference
PRAME	0.12	0.83	0.10	0.96	[9]
NPM1	0.29	0.90	0.45	0.98	[10]
SOX2	0.19	0.82	0.20	0.65	[12]
NFASC	0.08	0.32	0.05	0.24	[13]
MYCN	0.14	0.68	0.12	0.76	[14]
EHF	0.14	0.83	0.13	0.92	[15]
FAM60A	0.51	0.99	0.36	0.91	[16]
CD274	0.12	0.83	0.26	0.82	[17]
FGFR1	0. 16	0.90	0.20	0.90	[18]
STK11	0.39	1.00	0.42	0.96	[19]

Interestingly, our analysis shows a fraction of genes present low Pearson’s r of fitting and low ρ (Additional file 1: Figure S3D; Figure S3E), suggesting stable expression of these genes despite of CNV. To gain further insight into those genes (top 1000 genes with Pearson’s r of fitting from the lowest to the highest), Kyoto Encyclopedia of Genes and Genomes (KEGG) pathway analysis was carried out by utilizing the Database for Annotation, Visualization and Integrated Discovery (DAVID) [25]. Results demonstrated a strong enrichment of retinol metabolism, olfactory transduction, calcium signaling pathway, neuroactive ligand-receptor interaction, etc. (Additional file 2: Table S2; Table S3). Moreover, for a total number of 16,639 shared genes from cell lines and TCGA datasets, a high level of agreement on ρ was observed (Fig. 2c). Cell lines and tumors seem to exert similar trend. Wherein, 86.42% of shared genes showed a double-positive ρ among cell lines and TCGA datasets, while genes with a double-negative ρ only take up 1.63% in shared genes.

### Identification of genes with high concordance between CNV and differential gene expression

**Fig. 3:**
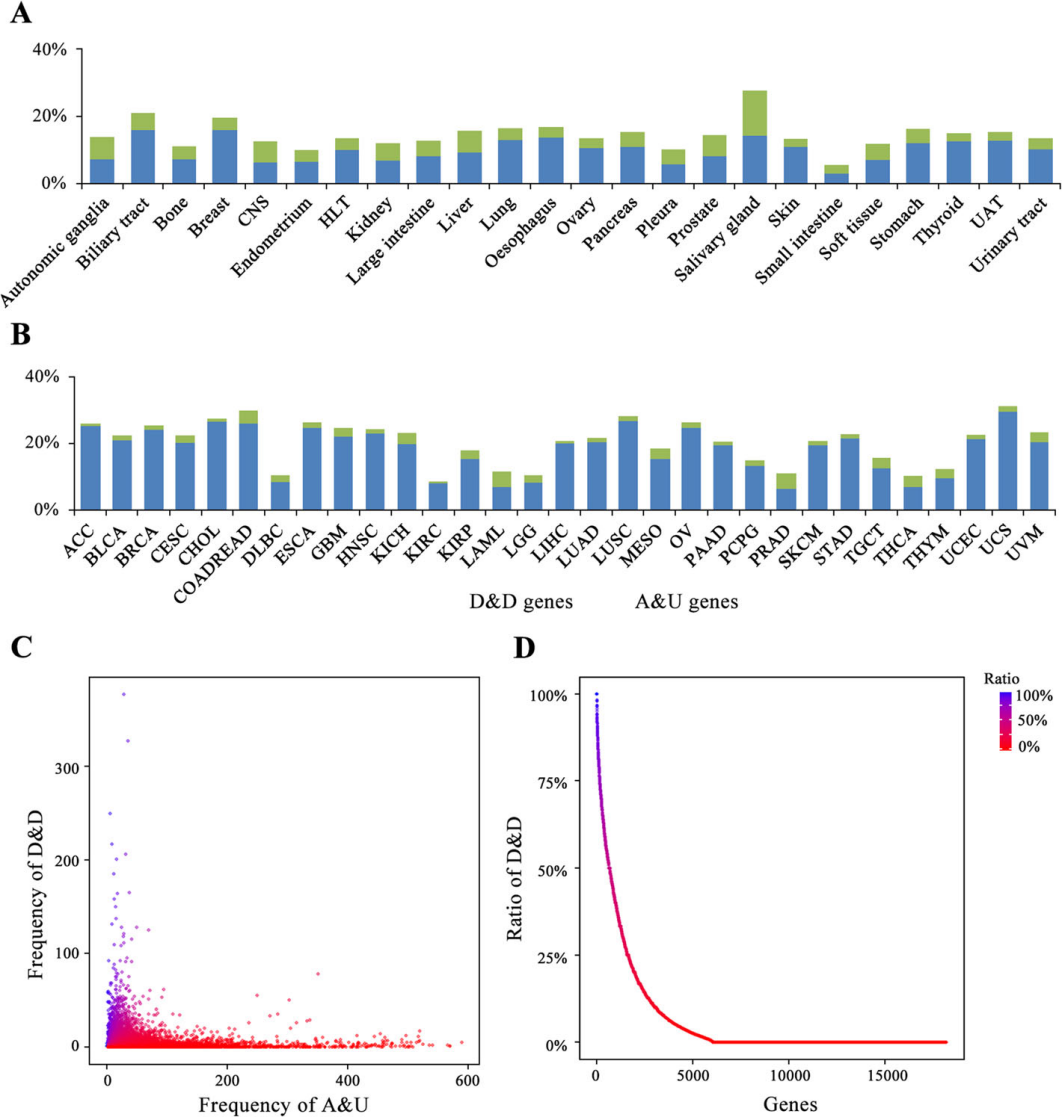
Comprehensive analysis of concordance between CNV and differential gene expression. **a.** The count ratio of copy number amplification and expression level upregulation (Blue), copy number deletion and expression level downregulation (Green) of the total variant copy number count across each primary site of cell lines in CCLE. CNS: central nervous system; HLT: haematopoietic and lymphoid tissue; UAT: upper aerodigestive tract. **b.** The count ratio of copy number amplification and expression level upregulation (Blue), copy number deletion and expression level downregulation (Green) of the total variant copy number count across each cancer type of TCGA. **c.** Scatter plot shows the distribution of 18,174 genes based on the frequency of copy number amplification and expression level upregulation (x-axis) and the frequency of copy number deletion and expression level downregulation (y-axis) among 9159 tumor samples in TCGA. The color represents the count ratio of copy number deletion and expression level downregulation against copy number amplification and expression level upregulation, blue representing higher ratio of copy number deletion and expression level downregulation, red representing higher ratio of copy number amplification and expression level upregulation. **d.** Genes were sorted according to the count ratio of copy number deletion and expression level downregulation against copy number amplification and expression level upregulation among 9159 tumor samples in TCGA. A&U: copy number amplification and expression level upregulation. D&D: copy number deletion and expression level downregulation

Based on two variation trend of copy number (amplification and deletion) and expression level (upregulation and downregulation), we divided variant trend into four classes: copy number amplification and expression level upregulation, copy number amplification and expression level downregulation, copy number deletion and expression level downregulation, copy number deletion and expression level upregulation (see Methods). We analyzed the frequencies of four variant trends across 24 primary sites of cell lines in CCLE database and 31 cancer types in TCGA. Intriguingly, the amount of copy number amplification and expression level upregulation, copy number deletion and expression level downregulation, representing our so-called highly concordant genes on CNV and differential gene expression, occupied 15 and 20% of the total variant copy number count on average for cell lines and tumor samples, respectively. Nevertheless, the mean proportion of copy number amplification and expression level downregulation, copy number deletion and expression level upregulation count only took up 0.7 and 0.5% for both cell lines and tumor samples (Additional file 1: Figure S4A and S4B), which indicated that the copy number amplification barely causes gene expression downregulation and the copy number deletion hardly promotes gene expression upregulation. It is obvious that the frequency of copy number amplification and expression level upregulation totally exceeded copy number deletion and expression level downregulation for both cell lines and tumor samples (Fig. 3a and b), which may be a result of selection on deletions [1].

Gene names in literature were transformed into official symbols. ρ: spearman’s correlation coefficient.

In order to identify highly concordant genes on CNV and differential gene expression, the sum of copy number amplification and expression level upregulation, copy number deletion and expression level downregulation for all genes across 9159 tumor samples in TCGA were counted (Fig. 3c). The red points close to x-axis owning a high count of copy number amplification and expression level upregulation and a low count of copy number deletion and expression level downregulation are preferentially defined as copy number amplified and expression level upregulated genes (AUGs), while the blue points close to y-axis owning a high count of copy number deletion and expression level downregulation and a low count of copy number amplification and expression level upregulation are regarded as copy number deleted and expression level downregulated genes (DDGs). Moreover, we observed that a majority of genes (83%, ratio < 0.1) belongs to AUGs (Fig. 3d). It may be caused by unknown selective pressures on amplification [1]. We attempt to revalidate the ten highly concordant genes through literature mining (9 AUGs, 1 DDG; shown in Table 2). Nine AUGs obtained preponderant count of copy number amplification and expression level upregulation compared to copy number deletion and expression level downregulation across 9159 tumor samples, while the only DDG (STK11) showed an overwhelming degree of copy number deletion and expression level downregulation (73%) against copy number amplification and expression level upregulation, in accordance with previous reports in literature (Table 2).

**Table 2:** Variation tendency revalidation of genes with a significant correlation between CNV and differential gene expression in literature

Official symbol	A&U	D&D	Variation tendency in literature	Reference
PRAME	19	0	↑	[9]
NPM1	40	0	↑	[10]
SOX2	303	0	↑	[12]
NFASC	44	0	↑	[13]
MYCN	24	0	↑	[14]
EHF	24	0	↑	[15]
FAM60A	96	0	↑	[16]
CD274	48	0	↑	[17]
FGFR1	113	0	↑	[18]
STK11	12	33	↓	[19]

Gene names in literature were transformed into official symbols. A&U represents the frequency of a gene with copy number amplified and expression level upregulated across 31 cancer types in TCGA, while D&D represents the frequency of a gene with copy number deleted and expression level downregulated. ↑ means the gene with copy number amplification and expression level upregulation in tumor, AUG; ↓ means the gene with copy number deletion and expression level downregulation in tumor, DDG.

**Fig. 4:**
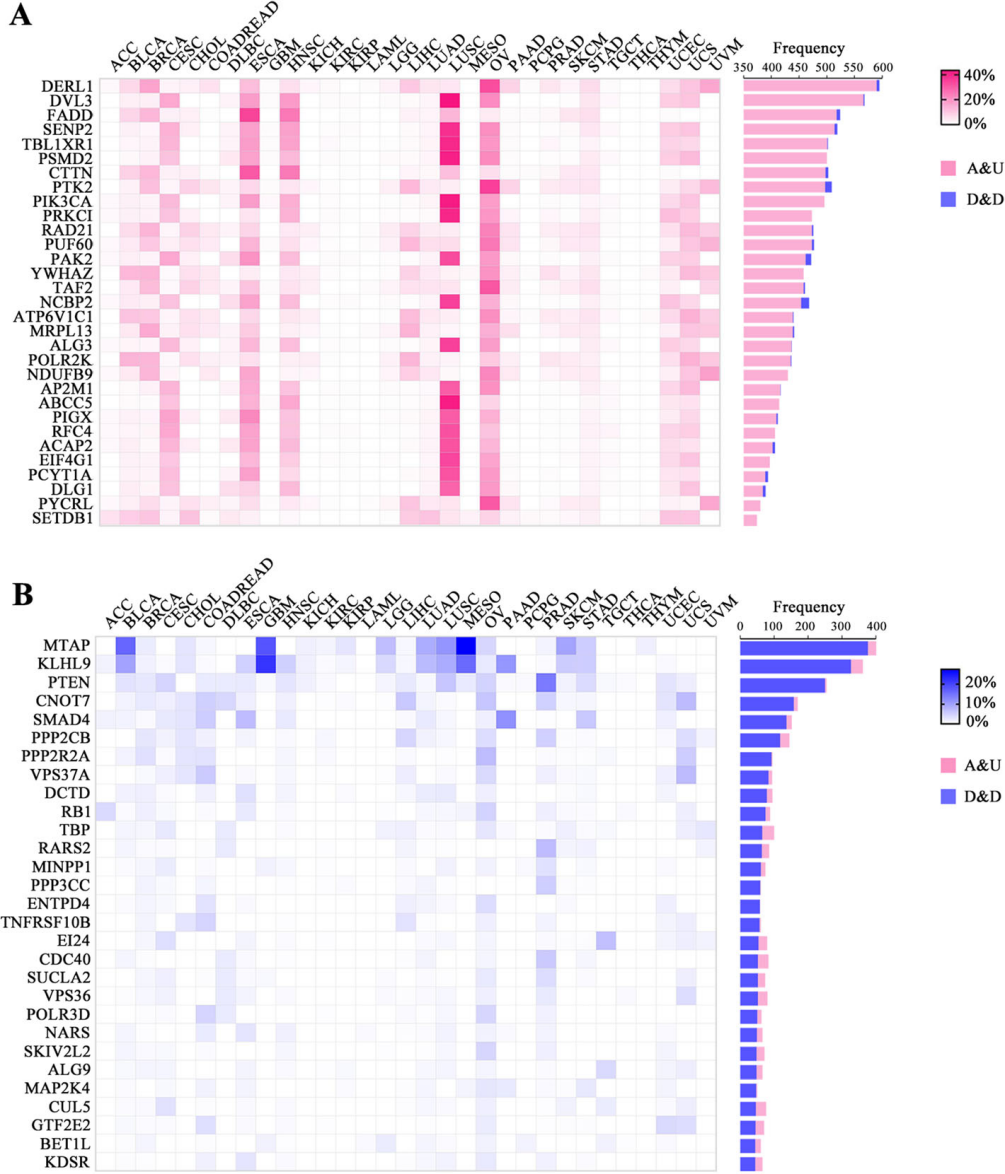
Identification of AUGs and DDGs across 31 various cancer types in TCGA. **a.** Representative AUGs (see Methods) were identified by the concordant rate of copy number amplification and expression level upregulation against copy number deletion and expression level downregulation and spearman correlation coefficient (the right barchart). The heatmap (left) shows the corresponding variant rate in each cancer type for each AUG. **b**. Representative DDGs were identified by the concordant rate of copy number deletion and expression level downregulation against copy number amplification and expression level upregulation and spearman correlation coefficient (the right barchart). The heatmap (left) shows the corresponding variant rate in each cancer type for each DDG. A&U: copy number amplification and expression level upregulation. D&D: copy number deletion and expression level downregulation

Thus, to identify AUGs and DDGs, the criteria applied in this work is the copy number amplification and expression level upregulation ratio against copy number deletion and expression level downregulation, copy number deletion and expression level downregulation ratio against copy number amplification and expression level upregulation with the cutoff value of 50 %. Further by filtering parameter included a higher ρ and a higher number of copy number amplification and expression level upregulation than the median level of 30 most popular oncogenes (Additional file 2: Table S4) or a higher number of copy number deletion and expression level downregulation than the median level of 10 tumor suppressor genes (Additional file 2:Table S5), which ultimately led to 560 AUGs such as DERL1, DVL3, FADD and 365 DDGs (e.g. MTAP, KLHL9, PTEN) (complete list in Additional file 2: Table S6). For representative AUGs and DDGs matched with KEGG pathway-related genes, their aberrant rate of copy number amplification and expression level upregulation, copy number deletion and expression level downregulation in corresponding tumor samples across 31 cancer types were analyzed and shown in Fig. 4a and Fig. 4b, respectively. Taken the AUG DERL1 as an example, this gene is amplified and overexpressed in a number of cancers, such as breast invasive carcinoma (BRCA, 16.84% patients), esophageal carcinoma (ESCA, 12.02% patients), liver hepatocellular carcinoma (LIHC, 10.17% patients), uterine carcinosarcoma (UCS, 10.71% patients), uveal melanoma (UVM, 16.25% patients), especially in ovarian serous cystadenocarcinoma (OV) with copy number amplification and expression level upregulation rate reaching 32.67% in 300 OV patients (Fig. 4a). Previous studies demonstrate DERL1 overexpression is significantly related to cancer cell proliferation, invasion and poor prognosis [26, 27]. Our study suggests this dysregulation may be driven by copy number amplification based on the extreme concordant correlation between copy number and gene expression. Figure 4b shows the distribution of copy number deletion and expression level downregulation rate across 31 cancers of 38 representative DDGs involving in many known tumor suppressor genes such as MTAP, KLHL9, PTEN, SMAD4, RB1, etc.

Additionally, we analyzed the distribution of AUGs and DDGs across 22 chromosomes (Additional file 2: Table S7). The maximal proportion of highly concordant genes of AUGs and DDGs (21.62%) were located in chromosome 8, followed by chromosome 1 (16.54%), which is in coherence with the previous finding published on nature that chromosome 1 possesses the largest number of genes exerting a strong relevance to numerous diseases such as cancer, Alzheimer’s disease, Parkinson’s disease [28]. As expected, the smallest chromosome 21 acquired the fewest percentage of highly concordant genes with 0.11%.

### AUGs and DDGs involved in proteolysis dysfunction in tumor

**Fig. 5:**
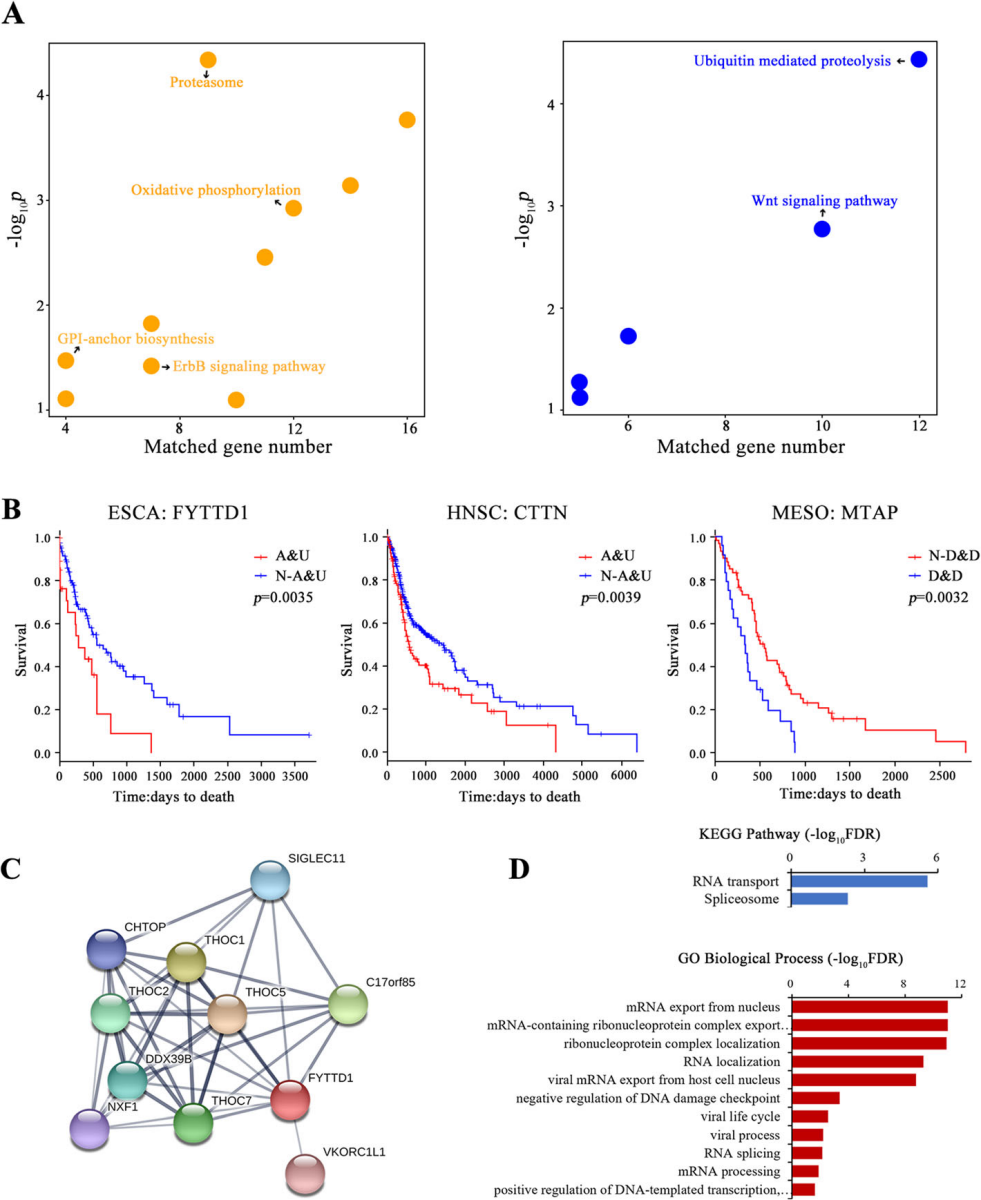
AUGs and DDGs are involved in different parts of cellular dysfunction in tumor. **a.** Scatter plot shows the KEGG enrichment of AUGs (left) and DDGs (right) by DAVID. The y-axis represents the *p* value and the x-axis represents the number of enriched genes. **b.** Kaplan-Meier curves for overall survival (OS) based on variation tendency of FYTTD1 in ESCA, CTTN in HNSC and MTAP in MESO. A&U represents samples with copy number amplification and gene expression upregulation, N-A&U represents samples without copy number amplification and gene expression upregulation; D&D represents samples with copy number deletion and gene expression downregulation, N-D&D represents samples without copy number deletion and gene expression downregulation. **c.** PPI network of interactive proteins with FYTTD1 by using STRING platform. **d.** Functional enrichments of the PPI network including GO biological process and KEGG pathways

To gain further insights into the function of AUGs and DDGs identified in this work, pathway enrichment analysis was performed on AUGs and DDGs by using DAVID. According to KEGG enrichment analysis results, a large proportion of AUGs were affiliated with metabolic pathways related to Oxidative phosphorylation and Glycosylphosphatidylinositol (GPI)-anchor biosynthesis (Fig. 5a), which manifest well-known gained function of metabolism-related proteins in tumors. In contrast, DDGs were significantly related with ubiquitin mediated proteolysis, wnt signaling pathway (Fig. 5a), whose dysfunction tend to lead to tumorigenesis [29], metastasis [30], resistance [31], etc. Interestingly, both AUGs and DDGs are remarkably enriched in the ubiquitin-proteasome system, which has been increasingly reported to be highly related to cancer recently [32, 33].

In addition, we found 9 highly concordant genes with a strong relation to patient overall survival including 5 AUGs and 1 DDG (Table 3). For examples, the copy number amplification and expression level upregulation of FYTTD1 and CTTN would remarkably cause a poor prognosis in tumor patients of ESCA and head and neck squamous cell carcinoma (HNSC) respectively (Fig. 5b), while the copy number deletion and expression level downregulation of MTAP was significantly associated with a worse prognosis (Fig. 5b). Wherein, it has been widely reported in literatures that five of these highly concordant genes are highly related with the development and progression of numerous cancers (See Additional file 2: Table S8 references) but FYTTD1. Thus, we used STRING platform [34] to analyze the protein-protein interaction (PPI) relationships of FYTTD1 (Fig. 5c). By integrated the functional enrichment results of KEGG pathways and Gene Ontology (GO) biological process of the PPI network (Fig. 5d), we found FYTTD1 plays an important role in RNA splicing in close relation to tumorigenesis, which may be a potential prognostic marker in ESCA.

**Table 3:** Nine highly concordant genes of 5 AUGs and 1 DDG show the strong association with clinical overall survival (OS) in corresponding cancer patients

Cancer	Official symbol	Class	*P* value
ESCA	FYTTD1	AUG	0.0035
ESCA	CTTN	AUG	0.0104
ESCA	PPFIA1	AUG	0.0307
ESCA	PIGX	AUG	0.0354
ESCA	FADD	AUG	0.0391
HNSC	CTTN	AUG	0.0039
HNSC	FADD	AUG	0.0087
HNSC	PPFIA1	AUG	0.0230
MESO	MTAP	DDG	0.0032

### Validation studies of correlation between CNV and differential gene expression on another independent dataset, the COSMIC cell lines project (CCLP)

**Fig. 6:**
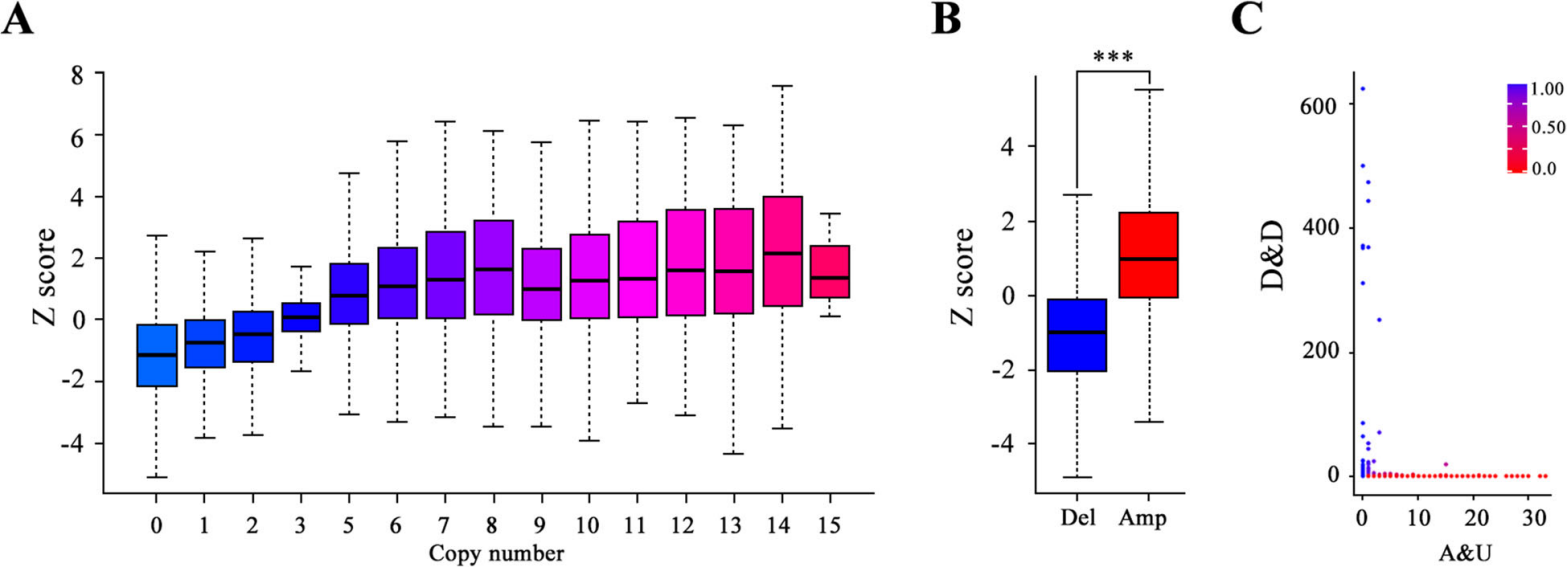
Validation studies of correlation between CNV and differential gene expression on CCLP. **a**. Boxplots show the relationship of Z score (y-axis) versus copy number value (x-axis, processed by PICNIC) among 1020 cell lines. **b.** The significant difference between Z scores of copy number amplified genes versus copy number deleted genes. **c.** Scatter plot shows the distribution of genes based on the frequency of copy number amplification and expression level upregulation (x-axis) and the frequency of copy number deletion and expression level downregulation (y-axis). The color represents the count ratio of copy number deletion and expression level downregulation against copy number amplification and expression level upregulation, blue representing higher ratio of copy number deletion and expression level downregulation, red representing higher ratio of copy number amplification and expression level upregulation. A&U: copy number amplification and expression level upregulation. D&D: copy number deletion and expression level downregulation

To validate our previous findings, the copy number and gene expression profiles were integrated across 1020 cell lines from CCLP, an independent dataset. Although CCLP utilized a different algorithm to calculate copy number variation, it still showed a positive correlation between copy number and expression level (Fig. 6a) by using a linear regression fitting on median Z score versus copy number (*r* = 0.89, *p* = 9.089e-6; Additional file 1: Fig. S5). In addition, gene expression levels with gene-wise copy number amplification exhibited a significant higher Z score than those with copy number deletion (Fig. 6b), in accordance with our results. Similarly, the amount of copy number amplification and expression level upregulation, copy number deletion and expression level downregulation occupied 28% of the total variant copy number count, while the proportion of copy number amplification and expression level downregulation, copy number deletion and expression level upregulation count only took up 1%. Thus, we counted the sum of copy number amplification and expression level upregulation, copy number deletion and expression level downregulation for the copy number aberrant genes among 1020 cell lines. Concordantly, most genes showed an overwhelming level of either copy number amplification and expression level upregulation or copy number deletion and expression level downregulation (93%, ratio > 0.9). Very fewer genes were with both high level of copy number amplification and expression level upregulation, copy number deletion and expression level downregulation (Fig. 6c). In addition, we overlapped the AUGs and DDGs identified by the ratio of copy number amplification and expression level upregulation versus copy number deletion and expression level downregulation between CCLP and TCGA respectively. Quite a number of highly concordant genes of AUGs (e.g. CTTN, GRB7, NSMCE2, KIAA0196, etc.) and DDGs (e.g. KLHL9, PTEN, PLAA, SMAD4, etc.) were shared within these two independent datasets. Collectively, these data suggested the close correlation between CNV and differential gene expression for most genes.

## Discussion

In this study, we provided strong evidences to support the high correlation between CNV and differential gene expression. This finding reveals the qualitative relationship between genetic variation and its downstream effect, especially for oncogenes and tumor suppressor genes, which is of a critical importance for prevention, diagnosis and treatment of cancer. First, by integrated analysis of CNV and differential gene expression of CCLE, NCI-60 and TCGA, it revealed a positive association between copy number and expression level with high Pearson’s r of fitting, positive ρ and significant *p* values. Besides, not only in cell lines but in patients copy number amplification strikingly harbored a higher expressed level compared to copy number deletion. Secondly, we investigated every gene over the relationship of CNV and differential gene expression across 9139 tumor samples and 1025 cell lines respectively. Our results showed the majority of genes the copy number displayed a positive linear influence on gene expression, indicating that genetic variation generated a direct effect on gene transcriptional level. In addition, we validated 10 genes with a significant correlation between CNV and differential gene expression through literature (Table 1). A strong correlation was confirmed combining ρ or Pearson’s r of fitting for 9 genes except the weak evidence for NFASC, possibly due to the difference of analytical method.

A recent study by GTEx consortium associated genetic variants with gene expression levels across 44 human healthy tissues and gene expression levels are found to be affected by local genetic variation for most genes based on eQTL analysis [24]. Meanwhile, it was reported that copy number and expression levels had a strong positive correlation for 99% abundantly expressed human genes by integrating predicted copy number and corrected expression level from 77,840 expression profiles [35]. Moreover, it has been widely reported that copy number is remarkably correlated with expression of protein in literature such as FGFR1 [36], HER2 [37, 38], MET [39], FADD [40], EGFR [37]. Message RNA, as intermediates between genes and functional proteins, plays a vital role in proteins production. Thus, we speculated gene expression might be correlated with protein expression as well for the high correlation and concordance between CNV and differential gene expression across cell lines and TCGA datasets and between CNV and differential protein expression of these five genes in literature (Additional file 2: Table S9, Table S10). Notably, FGFR1, known as fibroblast growth factor receptor 1, has been discovered that its copy number amplification is strikingly correlated with FGFR1 gene upregulation and FGFR1 protein upregulation in tumor samples [18, 36]. It indicated that the dysregulation of protein might attribute to original copy number aberrance through the concordant differential gene expression.

However, a fraction of genes’ expression level did have nothing to do with copy number keeping in a stable expression level over various copy number. We think these genes might be involved in the maintenance of the basal cellular function such as metabolism and signal transduction by the results of significant KEGG pathway enrichment including retinol metabolism, olfactory transduction, calcium signaling pathway, neuroactive ligand-receptor interaction, etc. (Additional file 2: Table S2 and Table S3). Otherwise, it has been well documented that 24% of the 575 housekeeping (HK) genes accounted for the metabolic proteins and 19% for RNA-interacting proteins [41]. Thus, we focus on the whole small nucleolar RNAs and found most genes were indeed expressed very stably versus CNV (Additional file 2: Table S11 and S12). Third, our results revealed the little existence of highly inconsonant genes of copy number amplification and expression level downregulation, copy number deletion and expression level upregulation (Additional file 1: Figure S4), which indicated that the copy number amplification barely causes gene expression downregulation and the copy number deletion hardly promotes gene expression upregulation. Otherwise, among the highly concordant genes with copy number amplification and expression level upregulation, copy number deletion and expression level downregulation, the frequency of copy number amplification and expression level upregulation evidently exceeded copy number deletion and expression level downregulation (Fig. 3b) possibly as a result of selection on deletions for it is unknown of the selective pressures on amplification [1]. We attempt to revalidate the ten highly concordant genes in literature (9 AUGs, 1 DDG; Table 2), whose results was highly consistent with the variation trend in literature.

Note that although the sample sizes of CCLE, NCI-60 and 31 cancers in TCGA were discrepant (Additional file 2: Table S1), they still showed a similar tendency of the association between CNV and differential gene expression (Fig. 1a; Additional file 1: Figure S1 and Figure. S2). Moreover, we observed a high level of agreement between cell lines and TCGA datasets which showed a consistent distribution of genes in Fig. 2a and Additional file 1: Fig. 3a including the ρ for 16,639 shared genes from (Fig. 2c) and a comparable Pearson’s r of fitting (Fig. 2b; Additional file 1: Figure S3C and Figure S3D). Our results suggested that this phenomenon was well conserved within cell lines and tissues.

In total, we identified 925 highly concordant genes including 560 AUGs and 365 DDGs. For examples, numerous studies reported that DERL1 overexpression was significantly related to cancer cell proliferation [26], invasion [42, 43] and poor prognosis [27], which might be driven by copy number amplification for DERL1 obtained the majority of copy number amplification and expression level upregulation in many cancers (Fig. 4a). Obviously, CNV-driven differentially expressed genes (DEGs) might broaden our insights into the mechanism of tumorigenesis, migration, resistance, poor prognosis, etc. for the increasing studies on CNV-driven DEGs [16, 44, 45]. In our study, a large proportion of AUGs were affiliated with metabolic pathways especially in terms of Oxidative phosphorylation and GPI-anchor biosynthesis (Fig. 5a), which suggested the gained function of metabolism-related proteins in tumors to provide more energy for cancer cells [46–48]. In contrast, DDGs were significantly related with ubiquitin mediated proteolysis and wnt signaling pathway (Fig. 5b), whose dysfunction tend to lead to tumorigenesis [29], metastasis [49], resistance [31], etc. With respect to wnt signaling, lost function of DDGs such as inhibitory SMAD4 and APC would definitely enhance the function of wnt signaling leading to tumorigenesis [50–57], while attenuated function of ubiquitin mediated proteolysis facilitate proliferation [58]. Wherein, we found 10 highly concordant genes with a strong relation to patient overall survival including 5 AUGs and 1 DDG (Table 3), while FYTTD1 has been hardly reported to be associated with cancer. By further integrated analysis of CNV and differential gene expression of FYTTD1 in ESCA patients, we observed that 24.73% patients showed a high level of copy number amplification with a median Z score of 4.15 which means FYTTD1 was strikingly overexpressed (Additional file 1: Figure S6). Wherein, AU patients occupied an overwhelming part among these 48 copy number amplified patients (84.44%) indicating global effect of CNV on FYTTD1 gene expression, which may be a potential driver gene or prognostic marker in ESCA. Therefore, highly concordant genes of AUGs and DDGs may provide new insights into the development and progression of cancer.

Additionally, we utilized another independent dataset (CCLP) to revalidate the relationship between CNV and differential gene expression. Although CCLP applied a different algorithm to calculate copy number variation, it also showed a positive correlation between copy number and expression level (Fig. 6a). Our results demonstrated that gene expression levels of copy number amplification substantially surpassed gene expression levels of copy number deletion (Fig. 6b). Besides, copy number amplification and expression level downregulation, copy number deletion and expression level upregulation versus copy number aberrant counts took up the smallest part (1%). Concordantly, most genes showed an overwhelming level of either copy number amplification and expression level upregulation or copy number deletion and expression level downregulation (93%, ratio > 0.9), and it was hardly existed of genes with both high level of copy number amplification and expression level upregulation, copy number deletion and expression level downregulation (Fig. 6c).

## Conclusions

In conclusion, this study demonstrated the close correlation between CNV and differential gene expression. Moreover, this trend is consistent across cell lines and patient samples. For the majority of genes, copy number shows a positive linear influence on gene expression (copy number amplification and expression level upregulation, copy number deletion and expression level downregulation), while copy number amplification barely causes gene expression downregulation and the copy number deletion hardly promotes gene expression upregulation. Furthermore, both AUGs and DDGs are remarkably enriched in the ubiquitin-proteasome system. In addition, we identified amplification and overexpression of FYTTD1 is highly related with poor prognosis in ESCA, which may be a potential prognostic marker in ESCA. Whereas, more in-depth studies are needed to further reveal molecular mechanisms between CNV and differential gene expression. Overall, it is of a critical importance to elucidate the relationship between copy number variation and gene expression for prevention, diagnosis and treatment of cancer.

## Supplementary information

**Additional file 1: Figure S1.** Integrative analysis of the association between differential gene expression and CNV across multiple cancer types. **Figure S2.** Integrative analysis of the association between differential gene expression and CNV across multiple cancer types. **Figure S3.** Most genes’ expression changes significantly correlated with their CNVs. A. Genes were sorted according to ρ on Z score versus copy number across 1025 cell lines, green representing a positive ρ and red meaning a negative one, representative oncogenes and tumor suppressor genes next to the corresponding points. B. An example of gene with copy number correlated with expression level in cell lines dataset. C. Histograms present the distribution of r for each genes based on the linear fitting results of median Z score with each variable copy number in cell lines datasets. D. Scatter plot shows the distribution of genes based on r of fitting (x-axis) and ρ (y-axis) for cell lines dataset (left) and TCGA dataset (right). E. An example of gene with copy number uncorrelated with expression level in cell lines dataset. **Figure S4.** The amount proportion of copy number amplification and expression level downregulation, copy number deletion and expression level upregulation versus the total variant copy number count across each primary site of cell lines in CCLE (A) and each cancer types of TCGA (B). CNS: central nervous system; HLT: haematopoietic and lymphoid tissue; UAT: upper aerodigestive tract. **Figure S5.** The linear regression fitting of median Z scores versus corresponding copy number among 1020 cell lines of CCLP. **Figure S6.** Integrated analysis of CNV and differential gene expression of FYTTD1 in ESCA patients.

**Additional file 2: Table S1.** Datasets of CCLE, NCI-60 and 31 cancers in TCGA and the number of samples used in this study. **Table S2.** KEGG pathways enrichment of top 1000 genes with r of fitting from the lowest to the highest for TCGA datasets. **Table S3.** KEGG pathways enrichment of top 1000 genes with r of fitting from the lowest to the highest for cell lines datasets combining CCLE and NCI60. **Table S4.** Lists of 30 most popular oncogenes with ρ and the count of copy number amplification and expression level upregulation across 9159 tumor samples. **Table S5.** Lists of 10 most popular tumor suppressor genes with ρ and the count of copy number deletion and expression level downregulation across 9159 tumor samples. **Table S6.** Identified 560 AUGs and 365 DDGs. **Table S7.** The distribution of AUGs and DDGs across 22 chromosomes. **Table S8.** High relation of 9 concordant genes with the development and progression of numerous cancers. **Table S9.** Correlation analysis of genes with a significant correlation between CNV and differential protein expression in literature. **Table S10.** Variation tendency validation of genes with a significant correlation between CNV and differential protein expression in literature. **Table S11.** Stable expression level over various copy number of small nucleolar RNAs in cell line dataset. **Table S12.** Stable expression level over various copy number of small nucleolar RNAs in T dataset.

## Data Availability

The datasets used during the current study are available in the cBioportal for Cancer Genomics (http://www.cbioportal.org/datasets, datasets names see Additional file 2: Table S1) and COSMIC Cell Lines Project (CCLP, v81, http://cancer.sanger.ac.uk/cell_lines). The accession numbers for the sequencing/copy number and transcriptional data of CCLP are, respectively, EGA: EGAS00001000978 and ArrayExpress: E-MTAB-3610. The datasets used and/or analysed during the current study available from the corresponding author on reasonable request.

## Abbreviations

AUG: Copy number amplified and expression level upregulated genes
BRCA: Breast invasive carcinoma
CCLE: Cancer cell line encyclopedia
CCLP: Cosmic cell lines project
CNV: Copy number variation
DAVID: Database for annotation, visualization and integrated discovery
DDG: Copy number deleted and expression level downregulated genes
ESCA: Esophageal carcinoma
GO: Gene ontology
HNSC: Head and neck squamous cell carcinoma
LIHC: Liver hepatocellular carcinoma
NSCLC: Non-small cell lung cancer
OS: Overall survival
OV: Ovarian serous cystadenocarcinoma
PPI: Protein-protein interaction
SNP: Single nucleotide polymorphism
STRING: Search tool for the retrieval of interacting genes
TCGA: The cancer genome atlas
UCS: Uterine carcinosarcoma
UVM: Uveal melanoma

## References

[1] Redon R, Ishikawa S, Fitch KR, Feuk L, Perry GH, Andrews TD (2006). Global variation in copy number in the human genome. Nature.

[2] Sachidanandam R, Weissman D, Schmidt SC, Kakol JM, Stein LD, Marth G (2001). A map of human genome sequence variation containing 1.42 million single nucleotide polymorphisms. Nature.

[3] International HapMap C (2003). The international HapMap project. Nature.

[4] Iafrate AJ, Feuk L, Rivera MN, Listewnik ML, Donahoe PK, Qi Y (2004). Detection of large-scale variation in the human genome. Nat Genet.

[5] Sebat J, Lakshmi B, Troge J, Alexander J, Young J, Lundin P (2004). Large-scale copy number polymorphism in the human genome. Science.

[6] Tuzun E, Sharp AJ, Bailey JA, Kaul R, Morrison VA, Pertz LM (2005). Fine-scale structural variation of the human genome. Nat Genet.

[7] Conrad DF, Andrews TD, Carter NP, Hurles ME, Pritchard JK (2006). A high-resolution survey of deletion polymorphism in the human genome. Nat Genet.

[8] Hinds DA, Kloek AP, Jen M, Chen X, Frazer KA (2006). Common deletions and SNPs are in linkage disequilibrium in the human genome. Nat Genet.

[9] Yang L, Wang YZ, Zhu HH (2017). Chang Y.

[10] Zhou C, Zhang W, Chen W, Yin Y, Atyah M, Liu S (2017). Integrated Analysis of copy number variations and gene expression profiling in hepatocellular carcinoma. Sci Rep.

[11] Huang YS, Liu WB, Han F, Yang JT, Hao XL, Chen HQ (2017). Copy number variations and expression of MPDZ are prognostic biomarkers for clear cell renal cell carcinoma. Oncotarget.

[12] Gut André, Moch Holger, Choschzick Matthias (2018). SOX2 Gene Amplification and Overexpression is Linked to HPV-positive Vulvar Carcinomas. International Journal of Gynecological Pathology.

[13] Samulin Erdem J, Arnoldussen YJ, Skaug V, Haugen A, Zienolddiny S (2017). Copy number variation, increased gene expression, and molecular mechanisms of neurofascin in lung cancer. Mol Carcinog.

[14] Kuzyk A, Booth S, Righolt C, Mathur S, Gartner J, Mai S (2015). MYCN overexpression is associated with unbalanced copy number gain, altered nuclear location, and overexpression of chromosome arm 17q genes in neuroblastoma tumors and cell lines. Genes Chromosomes Cancer.

[15] Shi J, Qu Y, Li X, Sui F, Yao D, Yang Q (2016). Increased expression of EHF via gene amplification contributes to the activation of HER family signaling and associates with poor survival in gastric cancer. Cell Death Dis.

[16] Dong G, Mao Q, Yu D, Zhang Y, Qiu M, Dong G (2017). Integrative analysis of copy number and transcriptional expression profiles in esophageal cancer to identify a novel driver gene for therapy. Sci Rep.

[17] Budczies J, Bockmayr M, Denkert C, Klauschen F, Groschel S, Darb-Esfahani S (2016). Pan-cancer analysis of copy number changes in programmed death-ligand 1 (PD-L1, CD274) - associations with gene expression, mutational load, and survival. Genes Chromosomes Cancer.

[18] Kwak Y, Nam SK, Seo AN, Kim DW, Kang SB, Kim WH (2015). Fibroblast growth factor receptor 1 gene copy number and mRNA expression in primary colorectal Cancer and its Clinicopathologic correlation. Pathobiology.

[19] Zhao N, Wilkerson MD, Shah U, Yin X, Wang A, Hayward MC (2014). Alterations of LKB1 and KRAS and risk of brain metastasis: comprehensive characterization by mutation analysis, copy number, and gene expression in non-small-cell lung carcinoma. Lung Cancer.

[20] Zhao M, Zhao Z (2016). Concordance of copy number loss and down-regulation of tumor suppressor genes: a pan-cancer study. BMC Genomics.

[21] Mermel CH, Schumacher SE, Hill B, Meyerson ML, Beroukhim R, Getz G (2011). GISTIC2.0 facilitates sensitive and confident localization of the targets of focal somatic copy-number alteration in human cancers. Genome Biol.

[22] Li B, Dewey CN (2011). RSEM: accurate transcript quantification from RNA-Seq data with or without a reference genome. BMC Bioinformatics.

[23] Greenman CD, Bignell G, Butler A, Edkins S, Hinton J, Beare D (2010). PICNIC: an algorithm to predict absolute allelic copy number variation with microarray cancer data. Biostatistics.

[24] Consortium GT, Laboratory DA, Coordinating Center -Analysis Working G, Statistical Methods groups-Analysis Working G, Enhancing Gg, Fund NIHC (2017). Genetic effects on gene expression across human tissues. Nature.

[25] Huang da W, Sherman BT, Lempicki RA (2009). Bioinformatics enrichment tools: paths toward the comprehensive functional analysis of large gene lists. Nucleic Acids Res.

[26] Tan X, He X, Jiang Z, Wang X, Ma L, Liu L (2015). Derlin-1 is overexpressed in human colon cancer and promotes cancer cell proliferation. Mol Cell Biochem.

[27] Dong Q, Fu L, Zhao Y, Tan S, Wang E (2017). Derlin-1 overexpression confers poor prognosis in muscle invasive bladder cancer and contributes to chemoresistance and invasion through PI3K/AKT and ERK/MMP signaling. Oncotarget.

[28] Gregory SG, Barlow KF, McLay KE, Kaul R, Swarbreck D, Dunham A (2006). The DNA sequence and biological annotation of human chromosome 1. Nature.

[29] Bashir T, Pagano M (2003). Aberrant ubiquitin-mediated proteolysis of cell cycle regulatory proteins and oncogenesis. Adv Cancer Res.

[30] Kramer N, Schmollerl J, Unger C, Nivarthi H, Rudisch A, Unterleuthner D (2017). Autocrine WNT2 signaling in fibroblasts promotes colorectal cancer progression. Oncogene.

[31] Lang Valérie, Aillet Fabienne, Xolalpa Wendy, Serna Sonia, Ceccato Laurie, Lopez-Reyes Rosa G., Lopez-Mato Maria Paz, Januchowski Radosław, Reichardt Niels-Christian, Rodriguez Manuel S. (2017). Analysis of defective protein ubiquitylation associated to adriamycin resistant cells. Cell Cycle.

[32] Ao N, Chen Q, Liu G (2017). The small molecules targeting ubiquitin-proteasome system for Cancer therapy. Comb Chem High Throughput Screen.

[33] Roeten MSF, Cloos J (2017). Jansen G.

[34] Szklarczyk D, Franceschini A, Wyder S, Forslund K, Heller D, Huerta-Cepas J (2015). STRING v10: protein-protein interaction networks, integrated over the tree of life. Nucleic Acids Res.

[35] Fehrmann RS, Karjalainen JM, Krajewska M, Westra HJ, Maloney D, Simeonov A (2015). Gene expression analysis identifies global gene dosage sensitivity in cancer. Nat Genet.

[36] Sousa V, Reis D, Silva M, Alarcao AM, Ladeirinha AF, d'Aguiar MJ (2016). Amplification of FGFR1 gene and expression of FGFR1 protein is found in different histological types of lung carcinoma. Virchows Arch.

[37] Jung MJ, Woo CG, Lee S, Chin S, Kim HK, Kwak JJ (2017). Gene copy number variation and protein overexpression of EGFR and HER2 in distal extrahepatic cholangiocarcinoma. Pathology.

[38] Lee MJ, Kim N, Choung HK, Choe JY, Khwarg SI, Kim JE (2016). Increased gene copy number of HER2 and concordant protein overexpression found in a subset of eyelid sebaceous gland carcinoma indicate HER2 as a potential therapeutic target. J Cancer Res Clin Oncol.

[39] Yin X, Zhang T, Su X, Ji Y, Ye P, Fu H (2015). Relationships between chromosome 7 gain, MET Gene Copy Number Increase and MET Protein Overexpression in Chinese Papillary Renal Cell Carcinoma Patients. PLoS One.

[40] Chien HT, Cheng SD, Chuang WY, Liao CT, Wang HM, Huang SF (2016). Clinical implications of FADD gene amplification and protein overexpression in Taiwanese Oral cavity squamous cell carcinomas. PLoS One.

[41] Eisenberg E, Levanon EY (2003). Human housekeeping genes are compact. Trends Genet.

[42] Klopfleisch R, Schutze M, Linzmann H, Brunnberg L, Gruber AD (2010). Increased Derlin-1 expression in metastases of canine mammary adenocarcinomas. J Comp Pathol.

[43] Dong QZ, Wang Y, Tang ZP, Fu L, Li QC, Wang ED (2013). Derlin-1 is overexpressed in non-small cell lung cancer and promotes cancer cell invasion via EGFR-ERK-mediated up-regulation of MMP-2 and MMP-9. Am J Pathol.

[44] Lu X, Ye K, Zou K, Chen J (2014). Identification of copy number variation-driven genes for liver cancer via bioinformatics analysis. Oncol Rep.

[45] Yang Z, Zhuan B, Yan Y, Jiang S, Wang T (2015). Integrated analyses of copy number variations and gene differential expression in lung squamous-cell carcinoma. Biol Res.

[46] Blucher C, Stadler SC (2017). Obesity and breast Cancer: current insights on the role of fatty acids and lipid metabolism in promoting breast Cancer growth and progression. Front Endocrinol (Lausanne).

[47] Iommarini L, Ghelli A, Gasparre G, Porcelli AM (1858). Mitochondrial metabolism and energy sensing in tumor progression. Biochim Biophys Acta.

[48] Liu Q, Luo Q, Halim A, Song G (2017). Targeting lipid metabolism of cancer cells: a promising therapeutic strategy for cancer. Cancer Lett.

[49] Puram SV, Tirosh I, Parikh AS, Patel AP, Yizhak K, Gillespie S, et al. Single-Cell Transcriptomic Analysis of Primary and Metastatic Tumor Ecosystems in Head and Neck Cancer. Cell. 2017. 10.1016/j.cell.2017.10.044 PMC5878932 29198524

[50] Pappas L, Xu XL, Abramson DH, Jhanwar SC (2017). Genomic instability and proliferation/survival pathways in RB1-deficient malignancies. Adv Biol Regul.

[51] Di Fiore R, D'Anneo A, Tesoriere G, Vento R (2013). RB1 in cancer: different mechanisms of RB1 inactivation and alterations of pRb pathway in tumorigenesis. J Cell Physiol.

[52] Ma J, Huang K, Ma Y, Zhou M, Fan S (2017). The TAZ-miR-224-SMAD4 axis promotes tumorigenesis in osteosarcoma. Cell Death Dis.

[53] Ahmed S, Bradshaw AD, Gera S, Dewan MZ, Xu R. The TGF-beta/Smad4 signaling pathway in pancreatic carcinogenesis and its clinical significance. J Clin Med. 2017;6:5. 10.3390/jcm6010005 PMC5294958 28067794

[54] Taniguchi K, Moroishi T, de Jong PR, Krawczyk M, Grebbin BM, Luo H (2017). YAP-IL-6ST autoregulatory loop activated on APC loss controls colonic tumorigenesis. Proc Natl Acad Sci U S A.

[55] Daly CS, Shaw P, Ordonez LD, Williams GT, Quist J, Grigoriadis A (2017). Functional redundancy between Apc and Apc2 regulates tissue homeostasis and prevents tumorigenesis in murine mammary epithelium. Oncogene.

[56] ElHefnawi M, Soliman B, Abu-Shahba N, Amer M (2013). An integrative meta-analysis of microRNAs in hepatocellular carcinoma. Genomics Proteomics Bioinformatics.

[57] Luna Coronell JA, Sergelen K, Hofer P, Gyurjan I (2018). The Immunome of Colon Cancer: functional in Silico Analysis of antigenic proteins deduced from IgG microarray profiling. Genomics Proteomics Bioinformatics.

[58] Mofers A, Pellegrini P, Linder S, D’Arcy P. Proteasome-associated deubiquitinases and cancer. Cancer Metastasis Rev. 2017. 10.1007/s10555-017-9697-6 PMC5721125 29134486

