# Supplementary material for: Copy number variation is highly correlated with differential gene expression: a pan-cancer study
Source: BMC Med Genet. 2019 Nov 9;20:175. doi: 10.1186/s12881-019-0909-5 (PMC6842483; doi:10.1186/s12881-019-0909-5)
Supplement: Supplementary file 1 — Additional file 1: Figure S1. Integrative analysis of the association between differential gene expression and CNV across multiple cancer types. Figure S2. Integrative analysis of the association between differential gene expression and CNV across multiple cancer types. Figure S3. Most genes’ expression changes significantly correlated with their CNVs. A. Genes were sorted according to ρ on Z score versus copy number across 1025 cell lines, green representing a positive ρ and red meaning a negative one, representative oncogenes and tumor suppressor genes next to the corresponding points. B. An example of gene with copy number correlated with expression level in cell lines dataset. C. Histograms present the distribution of r for each genes based on the linear fitting results of median Z score with each variable copy number in cell lines datasets. D. Scatter plot shows the distribution of genes based on r of fitting (x-axis) and ρ (y-axis) for cell lines dataset (left) and TCGA dataset (right). E. An example of gene with copy number uncorrelated with expression level in cell lines dataset. Figure S4. The amount proportion of copy number amplification and expression level downregulation, copy number deletion and expression level upregulation versus the total variant copy number count across each primary site of cell lines in CCLE (A) and each cancer types of TCGA (B). CNS: central nervous system; HLT: haematopoietic and lymphoid tissue; UAT: upper aerodigestive tract. Figure S5. The linear regression fitting of median Z scores versus corresponding copy number among 1020 cell lines of CCLP. Figure S6. Integrated analysis of CNV and differential gene expression of FYTTD1 in ESCA patients. [file 12881_2019_909_MOESM1_ESM.docx]

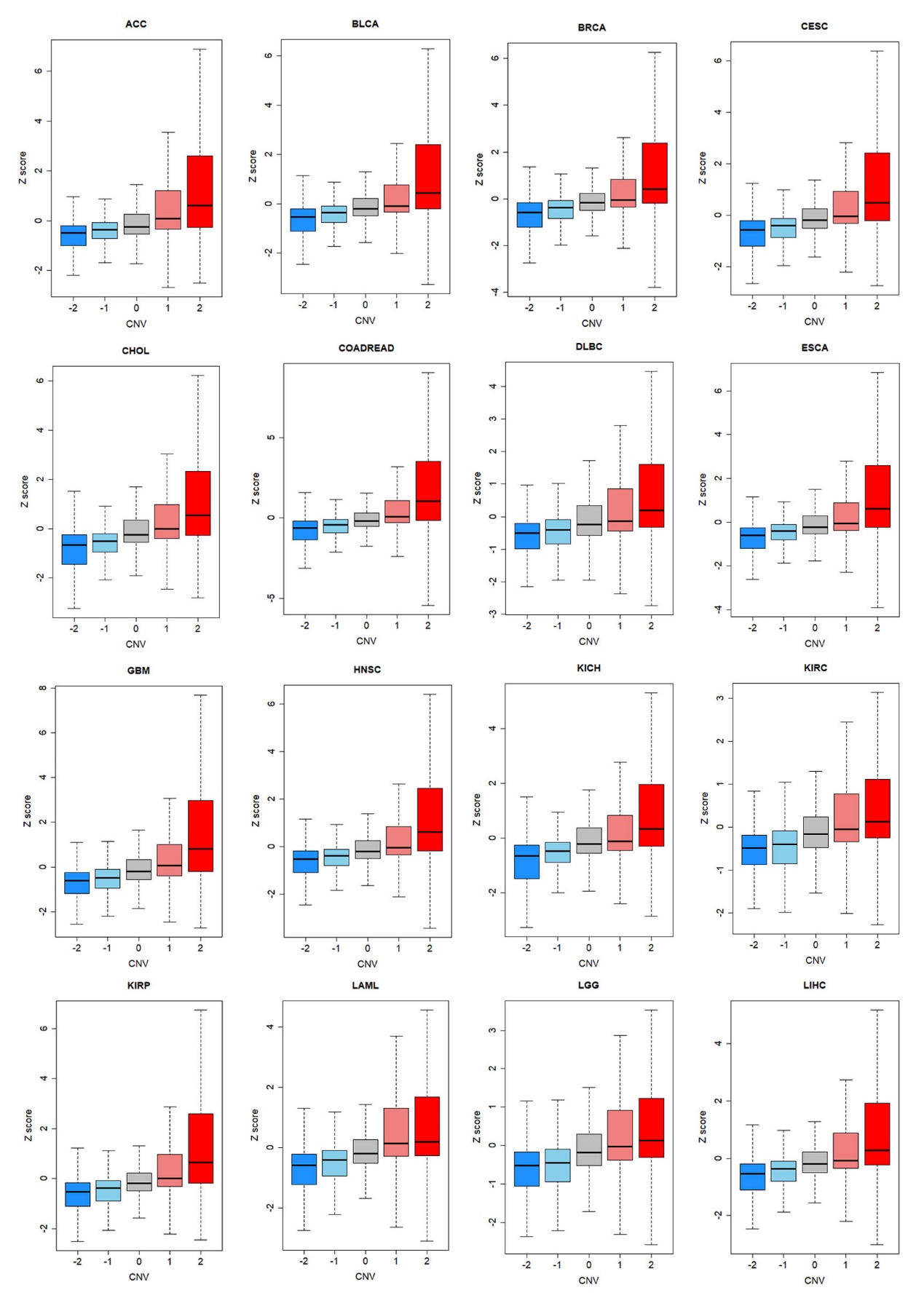


**Figure S1.** **Integrative analysis of the association between differential gene expression and CNV across multiple cancer types.**


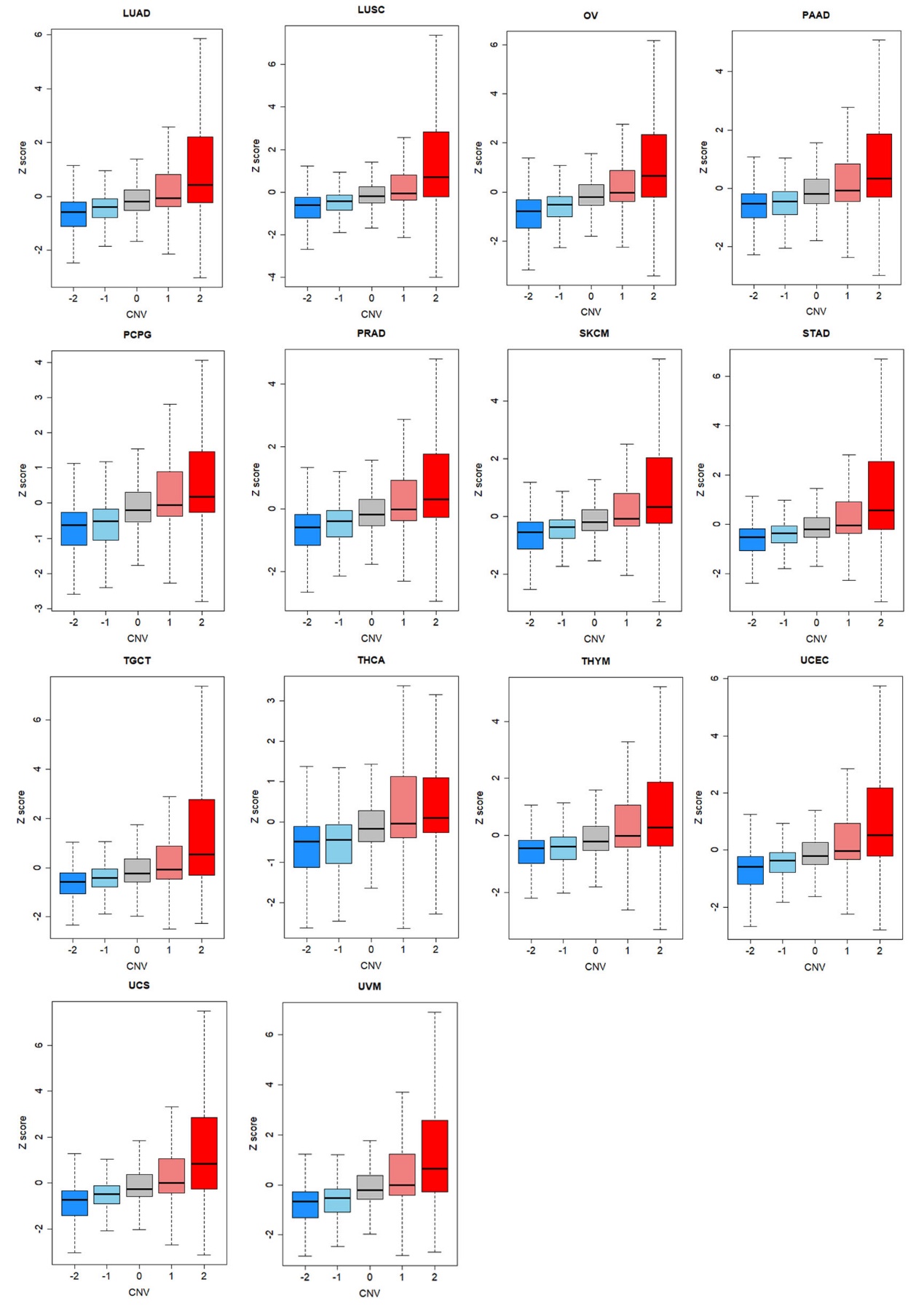


**Figure S2.** **Integrative analysis of the association between differential gene expression and CNV across multiple cancer types.**


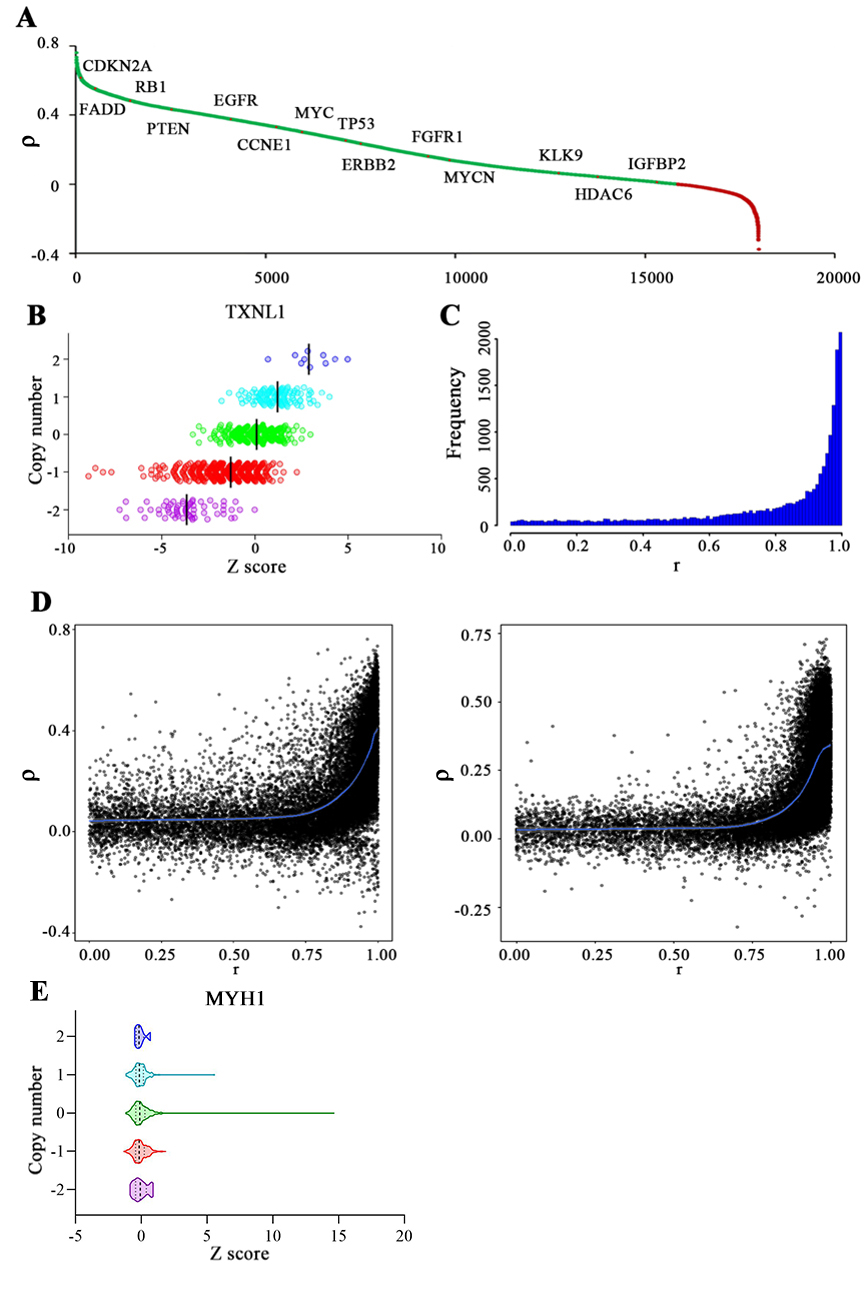


**Figure S3.** **Most genes’ expression changes significantly correlated with their CNVs. A.** Genes were sorted according to ρ on Z score versus copy number across 1025 cell lines, green representing a positive ρ and red meaning a negative one, representative oncogenes and tumor suppressor genes next to the corresponding points. **B.** An example of gene with copy number correlated with expression level in cell lines dataset. **C.** Histograms present the distribution of r for each genes based on the linear fitting results of median Z score with each variable copy number in cell lines datasets. **D.** Scatter plot shows the distribution of genes based on r of fitting (x-axis) and ρ (y-axis) for cell lines dataset (left) and TCGA dataset (right). **E.** An example of gene with copy number uncorrelated with expression level in cell lines dataset.


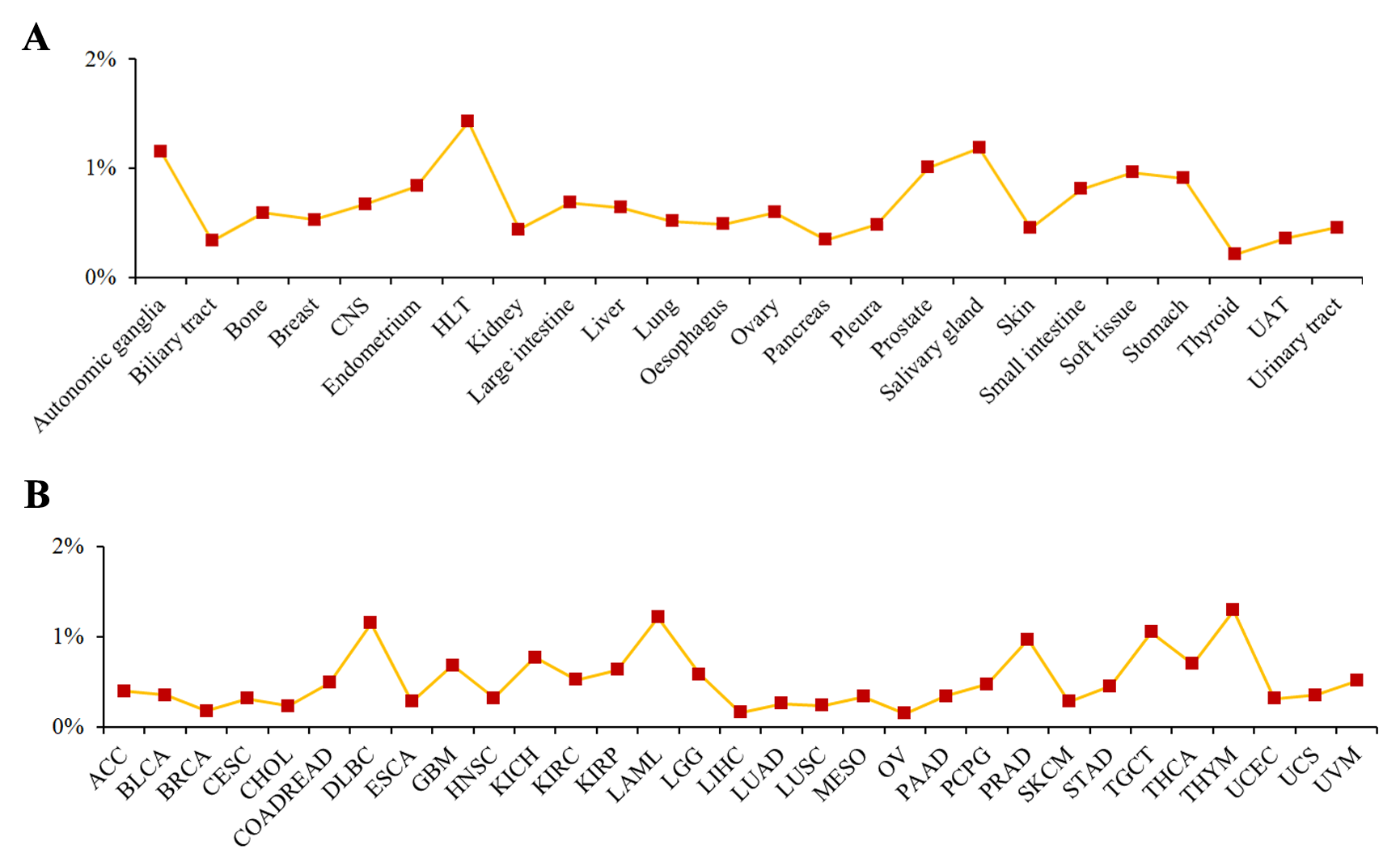


**Figure S4.** **The amount proportion of copy number amplification and expression level downregulation, copy number deletion and expression level upregulation versus the total variant copy number count across each primary site of cell lines in CCLE (A) and each cancer types of TCGA (B).** CNS: central nervous system; HLT: haematopoietic and lymphoid tissue; UAT: upper aerodigestive tract.


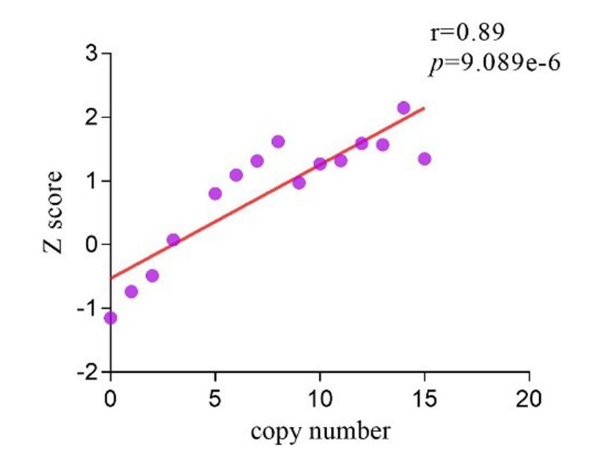


**Figure S5.** **The linear regression fitting of median Z scores versus corresponding copy number among 1020 cell lines of CCLP.**


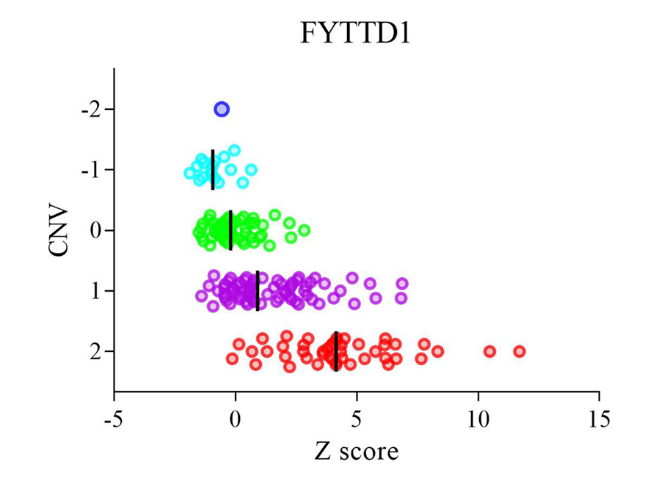


**Figure S6.** **Integrated analysis of CNV and differential gene expression of FYTTD1 in ESCA patients.**
