# Supplementary material for: Copy number variation is highly correlated with differential gene expression: a pan-cancer study
Source: BMC Med Genet. 2019 Nov 9;20:175. doi: 10.1186/s12881-019-0909-5 (PMC6842483; doi:10.1186/s12881-019-0909-5)
Supplement: Supplementary file 2 — Additional file 2: Table S1. Datasets of CCLE, NCI-60 and 31 cancers in TCGA and the number of samples used in this study. Table S2. KEGG pathways enrichment of top 1000 genes with r of fitting from the lowest to the highest for TCGA datasets. Table S3. KEGG pathways enrichment of top 1000 genes with r of fitting from the lowest to the highest for cell lines datasets combining CCLE and NCI60. Table S4. Lists of 30 most popular oncogenes with ρ and the count of copy number amplification and expression level upregulation across 9159 tumor samples. Table S5. Lists of 10 most popular tumor suppressor genes with ρ and the count of copy number deletion and expression level downregulation across 9159 tumor samples. Table S6. Identified 560 AUGs and 365 DDGs. Table S7. The distribution of AUGs and DDGs across 22 chromosomes. Table S8. High relation of 9 concordant genes with the development and progression of numerous cancers. Table S9. Correlation analysis of genes with a significant correlation between CNV and differential protein expression in literature. Table S10. Variation tendency validation of genes with a significant correlation between CNV and differential protein expression in literature. Table S11. Stable expression level over various copy number of small nucleolar RNAs in cell line dataset. Table S12. Stable expression level over various copy number of small nucleolar RNAs in T dataset. [file 12881_2019_909_MOESM2_ESM.docx]

**Table S1.** **Datasets of CCLE, NCI-60 and 31 cancers in TCGA and the number of samples used in this study.**

| Name | Datasets | Samples |
| --- | --- | --- |
| CCLE | Cancer Cell Line Encyclopedia (Novartis/Broad, Nature 2012) | 966 |
| NCI-60 | NCI-60 Cell Lines (NCI, Cancer Res 2012) | 59 |
| ACC | Adrenocortical Carcinoma (TCGA, Provisional) | 77 |
| BLCA | Bladder Urothelial Carcinoma (TCGA, Provisional) | 404 |
| BRCA | Breast Invasive Carcinoma (TCGA, Provisional) | 1075 |
| CESC | Cervical Squamous Cell Carcinoma and Endocervical Adenocarcinoma (TCGA, Provisional) | 292 |
| CHOL | Cholangiocarcinoma (TCGA, Provisional) | 36 |
| COADREAD | Colorectal Adenocarcinoma (TCGA, Provisional) | 376 |
| DLBC | Lymphoid Neoplasm Diffuse Large B-cell Lymphoma (TCGA, Provisional) | 48 |
| ESCA | Esophageal Carcinoma (TCGA, Provisional) | 183 |
| GBM | Glioblastoma Multiforme (TCGA, Provisional) | 147 |
| HNSC | Head and Neck Squamous Cell Carcinoma (TCGA, Provisional) | 514 |
| KICH | Kidney Chromophobe (TCGA, Provisional) | 66 |
| KIRC | Kidney Renal Clear Cell Carcinoma (TCGA, Provisional) | 525 |
| KIRP | Kidney Renal Papillary Cell Carcinoma (TCGA, Provisional) | 288 |
| LAML | Acute Myeloid Leukemia (TCGA, Provisional) | 166 |
| LGG | Brain Lower Grade Glioma (TCGA, Provisional) | 513 |
| LIHC | Liver Hepatocellular Carcinoma (TCGA, Provisional) | 364 |
| LUAD | Lung Adenocarcinoma (TCGA, Provisional) | 512 |
| LUSC | Lung Squamous Cell Carcinoma (TCGA, Provisional) | 498 |
| MESO | Mesothelioma (TCGA, Provisional) | 87 |
| OV | Ovarian Serous Cystadenocarcinoma (TCGA, Provisional) | 300 |
| PAAD | Pancreatic Adenocarcinoma (TCGA, Provisional) | 177 |
| PCPG | Pheochromocytoma and Paraganglioma (TCGA, Provisional) | 162 |
| PRAD | Prostate Adenocarcinoma (TCGA, Provisional) | 491 |
| SKCM | Skin Cutaneous Melanoma (TCGA, Provisional) | 367 |
| STAD | Stomach Adenocarcinoma (TCGA, Provisional) | 413 |
| TGCT | Testicular Germ Cell Cancer (TCGA, Provisional) | 150 |
| THCA | Thyroid Carcinoma (TCGA, Provisional) | 497 |
| THYM | Thymoma (TCGA, Provisional) | 119 |
| UCEC | Uterine Corpus Endometrial Carcinoma (TCGA, Provisional) | 176 |
| UCS | Uterine Carcinosarcoma (TCGA, Provisional) | 56 |
| UVM | Uveal Melanoma (TCGA, Provisional) | 80 |

**Table S2**. **KEGG pathways enrichment of top 1000 genes with r of fitting from the lowest to the highest for TCGA datasets.**

| Term | Genes | P-Value |
| --- | --- | --- |
| Olfactory transduction | 84 | 5.60E-28 |
| Retinol metabolism | 14 | 4.50E-05 |
| Hematopoietic cell lineage | 16 | 5.50E-05 |
| Metabolism of xenobiotics by cytochrome P450 | 14 | 1.80E-04 |
| Chemical carcinogenesis | 13 | 1.40E-03 |
| Drug metabolism - cytochrome P450 | 11 | 3.90E-03 |
| Cytokine-cytokine receptor interaction | 24 | 4.10E-03 |
| Malaria | 9 | 5.00E-03 |
| African trypanosomiasis | 7 | 8.60E-03 |
| Phagosome | 17 | 1.00E-02 |
| Hypertrophic cardiomyopathy (HCM) | 10 | 2.80E-02 |
| Staphylococcus aureus infection | 8 | 2.80E-02 |
| Primary immunodeficiency | 6 | 3.80E-02 |
| Steroid hormone biosynthesis | 8 | 4.00E-02 |
| Dilated cardiomyopathy | 10 | 4.20E-02 |

Significant pathways were filtered out with *p* < 0.05.

**Table S3.** **KEGG pathways enrichment of top 1000 genes with r of fitting from the lowest to the highest for cell lines datasets combining CCLE and NCI60.**

| Term | Genes | P-Value |
| --- | --- | --- |
| Neuroactive ligand-receptor interaction | 41 | 6.30E-05 |
| Calcium signaling pathway | 29 | 2.10E-04 |
| Dilated cardiomyopathy | 16 | 1.60E-03 |
| Serotonergic synapse | 19 | 1.80E-03 |
| Hematopoietic cell lineage | 16 | 1.80E-03 |
| Cytokine-cytokine receptor interaction | 31 | 2.70E-03 |
| Starch and sucrose metabolism | 9 | 2.80E-03 |
| PI3K-Akt signaling pathway | 41 | 5.00E-03 |
| Salivary secretion | 15 | 5.40E-03 |
| Cell adhesion molecules (CAMs) | 21 | 5.60E-03 |
| Asthma | 8 | 6.50E-03 |
| Jak-STAT signaling pathway | 21 | 7.10E-03 |
| Vascular smooth muscle contraction | 18 | 8.90E-03 |
| Carbohydrate digestion and absorption | 9 | 1.30E-02 |
| Amoebiasis | 16 | 1.50E-02 |
| Hypertrophic cardiomyopathy (HCM) | 13 | 1.50E-02 |
| Axon guidance | 18 | 1.70E-02 |
| Morphine addiction | 14 | 2.10E-02 |
| Fat digestion and absorption | 8 | 2.70E-02 |
| Nicotine addiction | 8 | 3.00E-02 |
| Cocaine addiction | 9 | 3.10E-02 |
| Oxytocin signaling pathway | 20 | 3.30E-02 |
| ECM-receptor interaction | 13 | 3.30E-02 |
| alpha-Linolenic acid metabolism | 6 | 3.80E-02 |
| Arrhythmogenic right ventricular cardiomyopathy (ARVC) | 11 | 4.30E-02 |
| Retrograde endocannabinoid signaling | 14 | 4.40E-02 |
| Maturity onset diabetes of the young | 6 | 4.50E-02 |
| Arachidonic acid metabolism | 10 | 4.50E-02 |

Significant pathways were filtered out with *p* < 0.05.

**Table S4**. **Lists of 30 most popular oncogenes with ρ and the count of copy number amplification and expression level upregulation across 9159 tumor samples.**

| Oncogenes | ρ | A&U |
| --- | --- | --- |
| ABL1 | 0.41 | 25 |
| AKT1 | 0.53 | 56 |
| ASXL1 | 0.43 | 144 |
| ATR | 0.47 | 201 |
| BRAF | 0.28 | 52 |
| CCND1 | 0.33 | 378 |
| CCNE1 | 0.29 | 208 |
| CDC73 | 0.45 | 149 |
| CDK12 | 0.36 | 180 |
| CDK4 | 0.36 | 125 |
| CDK9 | 0.48 | 38 |
| CTTN | 0.50 | 498 |
| DVL3 | 0.62 | 566 |
| EGFR | 0.25 | 252 |
| ERBB2 | 0.41 | 293 |
| FADD | 0.50 | 518 |
| FGFR1 | 0.20 | 113 |
| FOS | 0.10 | 6 |
| GNAS | 0.40 | 123 |
| KRAS | 0.40 | 180 |
| MCL1 | 0.28 | 132 |
| MDM2 | 0.40 | 181 |
| MET | 0.23 | 68 |
| MYC | 0.26 | 184 |
| MYCN | 0.12 | 24 |
| PIK3CA | 0.48 | 496 |
| PIK3CB | 0.42 | 118 |
| RAF1 | 0.65 | 85 |
| SOX2 | 0.20 | 303 |
| SRC | 0.35 | 76 |

A&U: copy number amplification and expression level upregulation. ρ: spearman's correlation coefficient.

**Table S5**. **Lists of 10 most popular tumor suppressor genes with ρ and the count of copy number deletion and expression level downregulation across 9159 tumor samples.**

| Tumor suppressor genes | ρ | D&D |
| --- | --- | --- |
| APC | 0.38825669 | 20 |
| ATM | 0.3252008 | 1 |
| CDKN2A | 0.3297812 | 30 |
| MEN1 | 0.3951002 | 4 |
| MLH1 | 0.5012401 | 9 |
| PTEN | 0.4859101 | 250 |
| RB1 | 0.45472758 | 75 |
| SMAD4 | 0.5849539 | 137 |
| TP53 | 0.31648386 | 17 |
| VHL | 0.42688062 | 3 |

D&D: copy number deletion and expression level downregulation. ρ: spearman's correlation coefficient.

**Table S6**. **Identified 560 AUGs and 365 DDGs.**

| AUGs | DDGs |
| --- | --- |
| DERL1 | MTAP |
| ZNF639 | KLHL9 |
| ACTL6A | PTEN |
| DVL3 | CHMP7 |
| ORAOV1 | MCPH1 |
| TRMT12 | CCDC25 |
| ABCF3 | ATAD1 |
| SENP5 | FOCAD |
| PPFIA1 | INTS9 |
| FADD | CNOT7 |
| ZNF7 | ELP3 |
| SENP2 | SMAD4 |
| MFN1 | XPO7 |
| EIF2B5 | PLAA |
| TATDN1 | MED4 |
| DCUN1D1 | TTI2 |
| TBL1XR1 | DCTN6 |
| PSMD2 | PPP2CB |
| CTTN | CAAP1 |
| PTK2 | ERICH1 |
| PIK3CA | INTS10 |
| DCAF13 | FBXO25 |
| C8ORF76 | WBP4 |
| FXR1 | PPP2R2A |
| LSG1 | CDADC1 |
| NSMCE2 | MTMR9 |
| KIAA0196 | VPS26B |
| HSF1 | TRIM35 |
| PRKCI | VPS37A |
| RAD21 | TRAPPC11 |
| PUF60 | LEPROTL1 |
| YEATS2 | DCTD |
| EMC2 | GPBP1 |
| PHF20L1 | UFSP2 |
| CHRAC1 | IFT74 |
| PAK2 | RB1 |
| UTP23 | CTDP1 |
| MAF1 | C11ORF57 |
| OPA1 | TNKS |
| YWHAZ | FAM160B2 |
| TAF2 | BIN3 |
| FAM91A1 | LYRM2 |
| ZC3H3 | R3HCC1 |
| MYNN | TBP |
| NCBP2 | ATP9B |
| TBCCD1 | RARS2 |
| PARL | MRPS31 |
| RNF139 | IST1 |
| ZNF696 | PCM1 |
| FYTTD1 | MINPP1 |
| ZNF706 | NUFIP1 |
| MAGEF1 | ESD |
| ATP6V1C1 | PPP3CC |
| MRPL13 | COG3 |
| ALG3 | ENTPD4 |
| POLR2K | IRF2 |
| PHC3 | TNFRSF10B |
| NUDCD1 | TRIM13 |
| FAM49B | ELAC1 |
| NDUFB9 | STK25 |
| ZNF707 | EI24 |
| VPS8 | NFRKB |
| SLC25A32 | RWDD4 |
| C8ORF33 | RAD17 |
| FAM131A | ITM2B |
| EBAG9 | BAP1 |
| TMEM41A | CDC40 |
| ZNF623 | BUD13 |
| AP2M1 | SUCLA2 |
| MRPL47 | VPS36 |
| ZNF16 | HDLBP |
| ABCC5 | RIC8A |
| KLHL24 | UFM1 |
| PIGX | MBD1 |
| GRINA | MEX3C |
| PABPC1 | TIMM21 |
| RFC4 | POLR3D |
| DNAJB11 | KPNA3 |
| ACAP2 | TXNL1 |
| EIF4G1 | ATXN10 |
| WDYHV1 | SYNCRIP |
| AZIN1 | NARS |
| ECT2 | SKIV2L2 |
| SCRIB | RNLS |
| PVT1 | ALG9 |
| SHARPIN | TBRG1 |
| PCYT1A | SPRYD7 |
| DNAJC19 | RTF1 |
| WDR53 | VPS39 |
| ENY2 | MRPS27 |
| MTDH | MAP2K4 |
| ATP11B | SNX14 |
| DLG1 | ZNF395 |
| PYCRL | CUL5 |
| FAM83H | 2-Sep |
| COMMD5 | GTF2E2 |
| ZNF250 | RNF214 |
| TMEM65 | BET1L |
| TIGD5 | KDSR |
| SETDB1 | SMIM15 |
| SLC52A2 | PGGT1B |
| SEC62 | AKIRIN2 |
| DEDD | ERI1 |
| STK3 | UBE4A |
| VPS72 | SETDB2 |
| DSCC1 | FXR2 |
| TBC1D31 | SMAD2 |
| MED30 | VPS4B |
| PPP1R2 | TBC1D22A |
| ZNF252P | BRD1 |
| UBR5 | QRICH1 |
| XXYLT1 | RPUSD4 |
| RNF168 | DHX38 |
| INTS8 | GIGYF2 |
| MCCC1 | SLC30A5 |
| ATAD2 | AGGF1 |
| PI4KB | RIOK2 |
| CPSF1 | FAM120B |
| UBXN7 | CLN8 |
| ASH2L | RMDN3 |
| GPAA1 | ZNF18 |
| CYC1 | NDUFA10 |
| TSNARE1 | SUGT1 |
| ZFP41 | ARIH2 |
| LRRC14 | CGGBP1 |
| TRAPPC9 | ATG12 |
| PRUNE | INTS6 |
| FBXO45 | MFAP1 |
| MTBP | BRD7 |
| SQLE | ZADH2 |
| ECE2 | DDX19A |
| NDUFB5 | PANK4 |
| LSM1 | CSDE1 |
| PDCD10 | COPS8 |
| BOP1 | CWC27 |
| RSRC1 | FAM35A |
| KPNA4 | MBTPS1 |
| WHSC1L1 | ELAC2 |
| AGO2 | ALG12 |
| POLR2H | CUL3 |
| TOP1MT | EIF4E2 |
| UBE2Q1 | USP4 |
| GMPS | WDR36 |
| BRD9 | ZNF202 |
| PTDSS1 | SNX19 |
| THEM6 | NHLRC3 |
| CYHR1 | NAA16 |
| ZNF251 | FBXO8 |
| VPS45 | ZUFSP |
| APH1A | KCTD9 |
| GFM1 | NAP1L4 |
| PIP5K1A | VPS11 |
| CLPTM1L | ATMIN |
| LYSMD1 | MAP1LC3B |
| EIF3H | ZC3H18 |
| ARHGAP39 | PPP6R2 |
| IGHMBP2 | DIS3L2 |
| ZNF34 | RHOA |
| CLCN2 | SPCS3 |
| BRF2 | DHX29 |
| TRA2B | RPL23AP53 |
| ZNF687 | TMEM218 |
| B4GALT3 | NUDT15 |
| CCDC127 | UBE3A |
| KIAA1429 | EMC8 |
| EIF4A2 | COPS7B |
| YY1AP1 | SHQ1 |
| USP21 | ORC3 |
| DDHD2 | MAP3K7 |
| DHX36 | CCNC |
| PROSC | C6ORF120 |
| NDUFAF6 | C10ORF76 |
| LAPTM4B | PPP2R2D |
| ANKRD46 | SPG11 |
| MRPL9 | PSMD7 |
| EHHADH | KARS |
| EXOC3 | DYM |
| FBXL6 | TMX3 |
| C8ORF82 | STK11 |
| PYGO2 | SMARCAL1 |
| TARS2 | ERCC8 |
| ERBB2 | C8ORF58 |
| NDUFS2 | RHOBTB2 |
| PPP1R16A | ZW10 |
| MROH1 | PUS3 |
| SMC4 | FAM192A |
| MIEN1 | POLR2C |
| ATP13A3 | HDHD2 |
| EIF3E | IWS1 |
| DGAT1 | ORC4 |
| ERLIN2 | PAPD4 |
| ADCK5 | ATP6V1B2 |
| URI1 | HINFP |
| SF3B4 | PHF11 |
| SDHC | NDUFAF1 |
| NSUN2 | RPAP1 |
| EXOSC4 | TLDC1 |
| VPS28 | MBD2 |
| TONSL | XRCC5 |
| SCAMP3 | FAM134A |
| RECQL4 | CDKN2AIP |
| RPRD2 | TCP1 |
| ENSA | BNIP3L |
| LRCH3 | TUBGCP2 |
| ARNT | TERF2 |
| TDRKH | PPWD1 |
| TM2D2 | RNASEH2B |
| RMDN1 | USP10 |
| STARD3 | DEF8 |
| CCT5 | SENP3 |
| ESRP1 | COX10 |
| SNX27 | ME2 |
| CHTOP | STRIP1 |
| PRCC | USP19 |
| POGZ | IP6K1 |
| COPA | AP3B1 |
| OTUD6B | MSH3 |
| HRSP12 | CHD1 |
| ZNF517 | REEP5 |
| C19ORF12 | SMIM8 |
| UBAP2L | TSPYL1 |
| PDCD6 | WTAP |
| POP4 | ZC3H13 |
| UFC1 | MTHFSD |
| PSMD4 | GLOD4 |
| UBQLN4 | SIKE1 |
| SLC33A1 | ARFIP1 |
| COMMD2 | PPIP5K2 |
| BAG4 | GOPC |
| GABPB2 | SIK3 |
| POGK | AQR |
| RPL8 | AP1G1 |
| SLC25A44 | MLYCD |
| DUSP12 | ANKRD11 |
| DNAJC21 | LINC00909 |
| GOLPH3L | WDR33 |
| CERS2 | FASTKD2 |
| SNAPIN | ACTR8 |
| GOLGA7 | UTP15 |
| COPS5 | GIN1 |
| SDHA | HDAC2 |
| PRPF3 | NUP43 |
| PIGM | TDRD3 |
| DPY19L4 | C15ORF57 |
| CRTC2 | HSDL1 |
| NCSTN | CENPBD1 |
| DAP3 | TCF25 |
| DROSHA | ZNF407 |
| PAPD7 | AP3D1 |
| WDR70 | PPP1R7 |
| F11R | DHX30 |
| DCAF8 | C3ORF38 |
| RAD1 | TAF9 |
| C1ORF43 | COL4A3BP |
| TADA1 | WDR41 |
| ZNF131 | KLLN |
| FLAD1 | MMS19 |
| EIF2A | INO80 |
| VDAC3 | CDAN1 |
| TCEB1 | OGFOD1 |
| RPL30 | VAC14 |
| CLK2 | TERF2IP |
| 6-Mar | CHMP1A |
| ILF2 | RNF167 |
| RAD54B | KIAA1468 |
| SCNM1 | NUP50 |
| NIT1 | CDPF1 |
| NMD3 | ERBB2IP |
| PSMB4 | FBXL17 |
| FASTKD3 | ARID1B |
| SEC61G | MLLT3 |
| POP1 | NIPA2 |
| GATAD2B | FAN1 |
| TMCO1 | LMF2 |
| C5ORF22 | ERCC3 |
| PAIP1 | ATG9A |
| YTHDF1 | WDR82 |
| CDC42SE1 | GLT8D1 |
| INTS4 | TMEM161B |
| MSTO1 | SRP19 |
| PSMD3 | SCAF8 |
| TIPRL | TMEM242 |
| POLR3C | DMTN |
| FAM189B | COX15 |
| HAX1 | FAM118B |
| ATF6 | LRCH1 |
| IFT80 | WDFY2 |
| MED10 | SNAP23 |
| TERF1 | CYB5B |
| NBN | SPG7 |
| PPP6R3 | MBP |
| ISG20L2 | CSNK1G2 |
| DAP | DDX20 |
| GID8 | MRPL44 |
| BRIX1 | NCL |
| GON4L | ATG16L1 |
| TMEM70 | RPP14 |
| UQCRB | PTCD2 |
| C5ORF51 | POC5 |
| FAM173B | SLC25A46 |
| ZFAND1 | DCP2 |
| METTL13 | SKP1 |
| GNPAT | SIRT3 |
| ADSS | EMC4 |
| OTUD7B | LCMT2 |
| ANP32E | GLG1 |
| ZC3H11A | ZDHHC7 |
| ARMC8 | PAFAH1B1 |
| GOLPH3 | CXXC1 |
| NIPBL | REXO1 |
| MED24 | CAPZA1 |
| KAT6A | STK16 |
| PEX19 | TRIP12 |
| RFWD2 | UBE2F |
| RAB2A | STX18 |
| STAU2 | RAB28 |
| RPS6KB1 | UBE2D3 |
| VAPB | ANKRA2 |
| TFDP2 | MTRF1L |
| ZNF622 | GLUD1 |
| SMIM19 | RPP30 |
| RRS1 | AP2A2 |
| ARHGEF11 | KBTBD6 |
| SH3BP5L | SRP14 |
| AP3M2 | IVD |
| C8ORF59 | TMEM87A |
| ORMDL3 | LRRC57 |
| PIGC | PITPNA |
| RBM34 | CTDNEP1 |
| AHCTF1 | WDR7 |
| FNTA | PIGN |
| MRPS28 | THAP4 |
| RAE1 | RNF123 |
| ADAR | TMEM115 |
| USF1 | CASP3 |
| CPNE3 | MIER3 |
| ATR | FAM172A |
| TLK2 | APC |
| SUPT5H | NT5DC1 |
| MOCS3 | PHF10 |
| RAB22A | HIF1AN |
| INTS3 | ACTR1A |
| GGPS1 | PHRF1 |
| TFB2M | DLAT |
| RNF13 | CEP164 |
| RUSC1 | HAUS2 |
| PPOX | EIF3J |
| SIAH2 | PIGB |
| MRPL36 | CSNK2A2 |
| CCT2 | CNTROB |
| SRP68 | TTC19 |
| TPM3 | TMEM167B |
| MRPL15 | CEPT1 |
| JTB | TRIM33 |
| OSGIN2 | RFT1 |
| CNOT2 | DIMT1 |
| GBA | IPO11 |
| NUP133 | EIF2AK4 |
| MTFR1 | VPS18 |
| RIPK2 | RNF111 |
| ALG8 | CHTF8 |
| HEATR6 | UBE2G1 |
| U2SURP | RABEP1 |
| IMPA1 | DVL2 |
| UQCRFS1 | USP16 |
| TAF4 |  |
| SPATA2 |  |
| PFDN2 |  |
| SCYL3 |  |
| TAF5L |  |
| NOL11 |  |
| CSTF1 |  |
| RTFDC1 |  |
| LSM14B |  |
| NUCKS1 |  |
| HPS3 |  |
| UBE2V2 |  |
| FAM104A |  |
| NELFCD |  |
| TOMM20 |  |
| UBE2V1 |  |
| TARS |  |
| GINS4 |  |
| PEX2 |  |
| SNAP47 |  |
| CEP72 |  |
| UBE2W |  |
| CLNS1A |  |
| CEP89 |  |
| DPM1 |  |
| RIT1 |  |
| ATP6V1H |  |
| TCEA1 |  |
| PAF1 |  |
| MRPS14 |  |
| POLB |  |
| MED1 |  |
| ANKRD27 |  |
| C1ORF131 |  |
| HLTF |  |
| TMEM68 |  |
| ARMC1 |  |
| MSL1 |  |
| METTL2A |  |
| LSM14A |  |
| OSBPL2 |  |
| C1ORF27 |  |
| KLHL12 |  |
| VCPIP1 |  |
| TRAM1 |  |
| SMG8 |  |
| SAMD4B |  |
| MED29 |  |
| CCT3 |  |
| DESI2 |  |
| COPB2 |  |
| SLC39A1 |  |
| NDUFS6 |  |
| KRAS |  |
| STAU1 |  |
| RSF1 |  |
| CCDC47 |  |
| ACBD6 |  |
| NUP107 |  |
| SS18L1 |  |
| TPD52L2 |  |
| SSR2 |  |
| MTG2 |  |
| ADIPOR1 |  |
| ARF1 |  |
| DBR1 |  |
| RAB11FIP1 |  |
| RNF121 |  |
| FTSJ3 |  |
| CCT6A |  |
| SAP30BP |  |
| RNF114 |  |
| YTHDF3 |  |
| FDPS |  |
| COG2 |  |
| ARFGEF1 |  |
| KDM2A |  |
| CASC3 |  |
| CLTC |  |
| GPATCH4 |  |
| IPO9 |  |
| SPRTN |  |
| TGS1 |  |
| LACTB2 |  |
| FAM86C1 |  |
| PSMD8 |  |
| CEBPG |  |
| BLZF1 |  |
| RB1CC1 |  |
| TUBD1 |  |
| METTL18 |  |
| MTRR |  |
| FBXL20 |  |
| GPATCH1 |  |
| TM9SF4 |  |
| MRGBP |  |
| HDGF |  |
| PPP1R15B |  |
| TBCE |  |
| USP32 |  |
| PAK4 |  |
| DIDO1 |  |
| RBBP5 |  |
| MTMR12 |  |
| NARS2 |  |
| UBA2 |  |
| PRPF6 |  |
| SLC25A36 |  |
| SERP1 |  |
| IMPAD1 |  |
| APPBP2 |  |
| AMZ2 |  |
| SMG7 |  |
| TROVE2 |  |
| C5ORF28 |  |
| ARPC1A |  |
| CHCHD7 |  |
| UCKL1 |  |
| PMF1 |  |
| RABIF |  |
| NADK2 |  |
| ZNF507 |  |
| DDX27 |  |
| GMEB2 |  |
| SLC50A1 |  |
| SMG5 |  |
| ARL8A |  |
| RNF170 |  |
| TACO1 |  |
| DDX59 |  |
| RNF7 |  |
| TIMM50 |  |
| EID2 |  |
| MRPS22 |  |
| IKBKB |  |
| NDUFC2 |  |
| WIPF2 |  |
| PSMD12 |  |
| HEATR1 |  |
| FH |  |
| MRPS30 |  |
| DDX42 |  |
| MTX1 |  |
| GORAB |  |
| ZKSCAN5 |  |
| THAP1 |  |
| PPP1CA |  |
| PRKRIR |  |
| DCAF7 |  |
| H3F3B |  |
| EXOC7 |  |
| UCK2 |  |
| FBXO28 |  |
| ZNF672 |  |
| MRPS17 |  |
| PFDN4 |  |
| MSL2 |  |
| PDAP1 |  |
| PTCD1 |  |
| COIL |  |
| SMARCD2 |  |
| POFUT1 |  |
| FBXO18 |  |
| IARS2 |  |
| PSMA7 |  |
| SUCO |  |
| TMEM183A |  |
| HOOK3 |  |
| INTS2 |  |
| ANGEL2 |  |
| PSMC5 |  |
| MRPS7 |  |
| PSMC4 |  |
| WDR26 |  |
| TAF6 |  |
| STRADA |  |
| FOXK2 |  |
| CDC73 |  |
| KDM5B |  |
| EXOC8 |  |
| SPIDR |  |
| NSMAF |  |
| TULP3 |  |
| MED13 |  |
| NUP85 |  |
| PDCD5 |  |
| ARFGAP1 |  |
| NPLOC4 |  |
| NUDT19 |  |
| DIEXF |  |
| RBM48 |  |
| RHNO1 |  |
| PDRG1 |  |
| MAPRE1 |  |
| STX16 |  |

**Table S7.** **The distribution of AUGs and DDGs across 22 chromosomes.**

| Chromosome | AUGs | DDGs | Sum |
| --- | --- | --- | --- |
| 1 | 144 | 9 | 153 |
| 2 | - | 27 | 27 |
| 3 | 87 | 18 | 105 |
| 4 | - | 13 | 13 |
| 5 | 35 | 41 | 76 |
| 6 | - | 26 | 26 |
| 7 | 9 | - | 9 |
| 8 | 162 | 38 | 200 |
| 9 | - | 7 | 7 |
| 10 | 1 | 15 | 16 |
| 11 | 17 | 28 | 45 |
| 12 | 6 | - | 6 |
| 13 | - | 28 | 28 |
| 14 | - |  | 0 |
| 15 | - | 27 | 27 |
| 16 | - | 34 | 34 |
| 17 | 43 | 16 | 59 |
| 18 | - | 25 | 25 |
| 19 | 22 | 4 | 26 |
| 20 | 34 | - | 34 |
| 21 | - | 1 | 1 |
| 22 | - | 8 | 8 |

- means null.

**Table S8**. **High relation of 9 concordant genes with the development and progression of numerous cancers.**

| Gene symbol | Reference |
| --- | --- |
| CTTN | [1] |
| PPFIA1 | [2] |
| PIGX | [3] |
| FADD | [4, 5] |
| MTAP | [6] |

**Table S9**. **Correlation analysis of genes with a significant correlation between CNV and differential protein expression in literature.**

| Protein | Gene symbol | Cell lines  ρ | Cell lines r | TCGA  ρ | TCGA r | Reference |
| --- | --- | --- | --- | --- | --- | --- |
| EGFR | EGFR | 0.38 | 0.95 | 0.25 | 0.74 | [7] |
| HER2 | ERBB2 | 0.23 | 0.80 | 0.41 | 0.76 | [7, 8] |
| FADD | FADD | 0.55 | 0.95 | 0.50 | 0.84 | [9] |
| FGFR1 | FGFR1 | 0.16 | 0.90 | 0.20 | 0.90 | [10] |
| MET | MET | 0.19 | 0.86 | 0.23 | 0.93 | [11] |

ρ: spearman's correlation coefficient.

**Table S10**. **Variation tendency validation of genes with a significant correlation between CNV and differential protein expression in literature.**

| Protein | Gene symbol | A&U | D&D | Variation tendency in literature | Reference |
| --- | --- | --- | --- | --- | --- |
| EGFR | EGFR | 252 | 0 | ↑ | [7] |
| HER2 | ERBB2 | 293 | 1 | ↑ | [7, 8] |
| FADD | FADD | 518 | 6 | ↑ | [9] |
| FGFR1 | FGFR1 | 113 | 0 | ↑ | [10] |
| MET | MET | 68 | 0 | ↑ | [11] |

A&U: copy number amplification and expression level upregulation. D&D: copy number deletion and expression level downregulation.

**Table S11. Stable expression level over various copy number of small nucleolar RNAs in cell line dataset.**

| Gene symbol | MZ  (CN = -2) | MZ  (CN = -1) | MZ  (CN = 0) | | MZ  (CN = 1) | MZ  (CN = 2) |
| --- | --- | --- | --- | --- | --- | --- |
| SNORA61 | -2.2914 | -0.4842 | 0.0728 | 0.3547 | | 1.7095 |
| SNORA74A | 0.2656 | -0.4576 | -0.1306 | -0.3566 | | -0.23525 |
| SNORA72 | -1.0622 | -0.5762 | 0.03055 | 0.5676 | | 1.24745 |
| SNORA25 | -1.5926 | -0.8316 | 0.0249 | 0.4344 | | 1.3091 |
| SNORD8 | -0.5458 | -0.31985 | -0.21325 | -0.1026 | | -0.13895 |
| SNORD114-3 | -0.3841 | -0.4486 | -0.4088 | -0.4353 | | -0.4965 |
| SNORA64 | -0.9393 | -0.7262 | -0.0521 | -0.08945 | | 0.6837 |
| SNORD104 | -1.8414 | -0.3899 | 0.0258 | 0.2707 | | 0.5986 |
| SNORA68 | -0.7618 | -0.5913 | -0.2001 | -0.08185 | | 0.403 |
| SNORA71B | 0.04875 | -0.1776 | -0.09955 | -0.0921 | | 0.11555 |

MZ represents median Z score of DGE. CN means copy number.

**Table S12. Stable expression level over various copy number of small nucleolar RNAs in T dataset.**

| Gene symbol | MZ  (CN = -2) | MZ  (CN = -1) | MZ  (CN = 0) | MZ  (CN = 1) | | MZ  (CN = 2) |
| --- | --- | --- | --- | --- | --- | --- |
| SNORA77  SNORA70  SNORA62  SNORA63  SNORA55  SNORA26  SNORA58  SNORA31  SNORA66  SNORA51  SNORA25  SNORA40  SNORA44  SNORA67  SNORA72  SNORA16B  SNORA36B  SNORA14B  SNORA64  SNORA75  SNORA12  SNORA70B  SNORA19  SNORA48  SNORA4  SNORA1  SNORA41  SNORA6  SNORA24  SNORA7B  SNORA18  SNORA81  SNORA11  SNORA57  SNORA47  SNORA13  SNORA74A  SNORA27  SNORA68  SNORA74B  SNORA38  SNORA8  SNORA20  SNORA29  SNORA5A  SNORA5B  SNORA5C  SNORA15  SNORA22  SNORA14A  SNORA32  SNORA30  SNORA84  SNORA65  SNORA52  SNORA54  SNORA23  SNORA34  SNORA2A  SNORA2B  SNORA53  SNORA9  SNORA49  SNORA79  SNORA46  SNORA11B  SNORA28  SNORA10  SNORA69  SNORA59B  SNORA21  SNORA38B  SNORA37  SNORA71B  SNORA71A  SNORA71C  SNORA71D  SNORA11D  SNORA36A | NA  -0.2218  1.6542  NA  -0.246  NA  NA  -0.2905  NA  -0.1151  -0.2097  -0.2918  0.0278  -0.6083  -0.4113  -0.1603  -0.116  -0.274  -0.1405  -0.1746  0.8386  -0.05595  NA  -0.2638  NA  -0.0866  -0.1687  -0.3815  NA  -0.482  3.0171  NA  -0.2018  NA  -0.2227  -0.1637  -0.2473  -0.2053  -0.1608  -0.2402  NA  NA  NA  -0.2584  -0.3498  -0.1998  -0.3612  NA  NA  -0.09525  -0.0789  NA  -0.2009  -0.3515  -0.3861  -0.1466  -0.3006  NA  -0.2334  -0.1229  -0.3416  -0.1865  -0.1986  NA  NA  -0.0692  -0.0946  -0.4767  -0.0888  -0.4489  NA  -0.16795  -0.1784  -0.3159  -0.2674  -0.2661  -0.1915  -0.1246  -0.1539 | -0.0914  -0.2218  -0.2605  -0.0194  -0.167  -0.1613  -0.0875  -0.2905  -0.0753  -0.1151  -0.2097  -0.2918  -0.1891  -0.6083  -0.4113  -0.1684  -0.0978  -0.2163  -0.1405  -0.1746  -0.2188  -0.0814  -0.0443  -0.2638  -0.1833  -0.0866  -0.1687  -0.3815  -0.4152  -0.4223  -0.394  -0.1015  -0.2018  -0.1613  -0.208  -0.1628  -0.31995  -0.2053  -0.1608  -0.2448  -0.1265  -0.8069  -0.2414  -0.2584  -0.2768  -0.1532  -0.2538  -0.1072  NA  -0.089  -0.0789  -0.0443  -0.1983  -0.4113  -0.2948  -0.1153  -0.1945  NA  -0.2107  -0.1388  -0.204  -0.1865  -0.142  -0.0902  -0.1817  -0.0781  -0.1034  -0.2795  -0.0888  -0.4801  -0.2977  -0.1414  -0.1784  -0.2601  -0.2714  -0.2209  -0.1814  -0.1146  -0.1434 | -0.0914  -0.2218  -0.2605  -0.2616  -0.167  -0.1613  -0.0875  -0.2905  -0.0753  -0.1151  -0.2097  -0.2918  -0.2202  -0.6083  -0.4113  -0.1591  -0.0933  -0.2056  -0.1405  -0.1746  -0.2188  -0.0814  -0.0443  -0.2638  -0.1833  -0.0866  -0.1687  -0.2758  -0.4152  -0.2192  -0.394  -0.1715  -0.2018  -0.11975  -0.2227  -0.1424  -0.1813  -0.2053  -0.1608  -0.1941  -0.1265  -0.1708  -0.2414  -0.2584  -0.279  -0.1289  -0.2612  -0.1072  -0.1508  -0.1015  -0.0789  -0.0443  -0.1866  -0.4087  -0.3091  -0.1354  -0.1945  -0.2515  -0.1945  -0.1388  -0.1461  -0.1865  -0.1567  -0.0902  -0.1817  -0.0781  -0.0946  -0.2585  -0.0888  -0.5209  -0.2977  -0.1528  -0.1572  -0.2575  -0.2482  -0.2176  -0.1747  -0.094  -0.137 | | -0.0914  -0.2218  -0.2605  -0.0679  -0.162  -0.1613  -0.0875  -0.2905  -0.0753  -0.1151  -0.2097  -0.2918  -0.1891  -0.6083  -0.4113  -0.1819  -0.0978  -0.1752  -0.1405  -0.1746  -0.2188  -0.0814  -0.0443  -0.2638  -0.1833  -0.0866  -0.1687  -0.2759  -0.4152  0.0567  -0.394  -0.1689  -0.2018  -0.13705  -0.2227  -0.1004  -0.1797  -0.2053  -0.1608  -0.129  -0.1265  -0.93525  -0.2414  -0.2584  -0.279  -0.1532  -0.2538  -0.1072  -0.1508  -0.1015  -0.0789  -0.0443  -0.1935  -0.3583  -0.2948  -0.1173  -0.1945  -0.2515  -0.1945  -0.14  -0.0796  -0.1865  -0.1683  -0.0902  -0.1817  -0.0827  -0.0833  -0.2081  -0.0888  -0.4946  -0.2977  -0.1245  -0.182  -0.2293  -0.2482  -0.2254  -0.1731  -0.094  -0.137 | -0.0914  -0.2218  NA  -0.2616  -0.167  -0.1613  NA  -0.2905  NA  -0.1151  -0.2097  -0.2918  -0.1744  0.61215  -0.4113  -0.1962  -0.11305  -0.1752  -0.1405  -0.1746  NA  -0.0814  NA  -0.2638  3.5659  NA  NA  -0.1942  NA  0.2232  -0.394  NA  NA  -0.1613  -0.2723  -0.0735  -0.202  -0.2053  -0.1608  -0.1941  NA  -0.4606  -0.2414  -0.26925  -0.2768  -0.1563  -0.2439  -0.1072  NA  -0.1015  NA  -0.0443  -0.1652  -0.3583  -0.257  -0.1466  -0.0975  NA  -0.2182  -0.1388  -0.07515  -0.1865  -0.1743  NA  NA  -0.0804  -0.0745  -0.2081  -0.0888  -0.3478  -0.2977  -0.1245  -0.1946  -0.2293  -0.1626  -0.1977  -0.1533  -0.1146  -0.137 |
| SNORA56 | -0.1724 | -0.1724 | -0.1672 | | -0.1672 | -0.1672 |

MZ represents median Z score of DGE. CN means copy number. NA is not available.

**References**

[1] Buday L, Downward J. Roles of cortactin in tumor pathogenesis. Biochim Biophys Acta 2007;1775:263-73.

[2] Chiaretti S, de Curtis I. Role of Liprins in the Regulation of Tumor Cell Motility and Invasion. Curr Cancer Drug Targets 2016;16:238-48.

[3] Nakakido M, Tamura K, Chung S, Ueda K, Fujii R, Kiyotani K, et al. Phosphatidylinositol glycan anchor biosynthesis, class X containing complex promotes cancer cell proliferation through suppression of EHD2 and ZIC1, putative tumor suppressors. Int J Oncol 2016;49:868-76.

[4] Bhojani MS, Chen G, Ross BD, Beer DG, Rehemtulla A. Nuclear localized phosphorylated FADD induces cell proliferation and is associated with aggressive lung cancer. Cell cycle 2005;4:1478-81.

[5] Shimada K, Nakamura M, Ishida E, Konishi N. Molecular roles of MAP kinases and FADD phosphorylation in prostate cancer. Histol Histopathol 2006;21:415-22.

[6] Bertino JR, Waud WR, Parker WB, Lubin M. Targeting tumors that lack methylthioadenosine phosphorylase (MTAP) activity: current strategies. Cancer Biol Ther 2011;11:627-32.

[7] Jung MJ, Woo CG, Lee S, Chin S, Kim HK, Kwak JJ, et al. Gene copy number variation and protein overexpression of EGFR and HER2 in distal extrahepatic cholangiocarcinoma. Pathology 2017;49:582-8.

[8] Lee MJ, Kim N, Choung HK, Choe JY, Khwarg SI, Kim JE. Increased gene copy number of HER2 and concordant protein overexpression found in a subset of eyelid sebaceous gland carcinoma indicate HER2 as a potential therapeutic target. J Cancer Res Clin Oncol 2016;142:125-33.

[9] Chien HT, Cheng SD, Chuang WY, Liao CT, Wang HM, Huang SF. Clinical Implications of FADD Gene Amplification and Protein Overexpression in Taiwanese Oral Cavity Squamous Cell Carcinomas. PLoS One 2016;11:e0164870.

[10] Sousa V, Reis D, Silva M, Alarcao AM, Ladeirinha AF, d'Aguiar MJ, et al. Amplification of FGFR1 gene and expression of FGFR1 protein is found in different histological types of lung carcinoma. Virchows Arch 2016;469:173-82.

[11] Yin X, Zhang T, Su X, Ji Y, Ye P, Fu H, et al. Relationships between Chromosome 7 Gain, MET Gene Copy Number Increase and MET Protein Overexpression in Chinese Papillary Renal Cell Carcinoma Patients. PLoS One 2015;10:e0143468.
